# Load-induced enhancement of Dynein force production by LIS1–NudE *in vivo* and *in vitro*

**DOI:** 10.1038/ncomms12259

**Published:** 2016-08-04

**Authors:** Babu J. N. Reddy, Michelle Mattson, Caitlin L. Wynne, Omid Vadpey, Abdo Durra, Dail Chapman, Richard B. Vallee, Steven P. Gross

**Affiliations:** 1 Department of Developmental and Cell Biology, University of California Irvine, Irvine, California 92697, USA; 2 Department of Pathology and Cell Biology, Columbia University, New York 10032, USA

## Abstract

Most sub-cellular cargos are transported along microtubules by kinesin and dynein molecular motors, but how transport is regulated is not well understood. It is unknown whether local control is possible, for example, by changes in specific cargo-associated motor behaviour to react to impediments. Here we discover that microtubule-associated lipid droplets (LDs) in COS1 cells respond to an optical trap with a remarkable enhancement in sustained force production. This effect is observed only for microtubule minus-end-moving LDs. It is specifically blocked by RNAi for the cytoplasmic dynein regulators LIS1 and NudE/L (Nde1/Ndel1), but not for the dynactin p150^*Glued*^ subunit. It can be completely replicated using cell-free preparations of purified LDs, where duration of LD force production is more than doubled. These results identify a novel, intrinsic, cargo-associated mechanism for dynein-mediated force adaptation, which should markedly improve the ability of motor-driven cargoes to overcome subcellular obstacles.

## Abstract (web summary)

Transport of large cargo through the cytoplasm can encounter physical impediments which should be overcome. Here the authors show that lipid droplets constrained by an optical trap respond with an increase in dynein-mediated force that is dependent on dynein regulators LIS1 and NudE/L, but not on p150^glued^.

## Graphical abstract

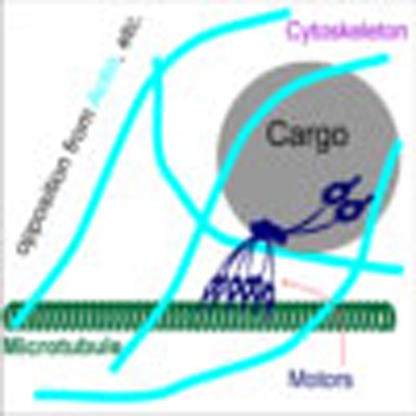

Microtubule-based molecular motors position cargos within the cytoplasm and differentially transport them within axons, to create sub-cellular order. This uses a limited number of plus-end (kinesin) and minus-end (dynein) molecular motors per cargo^1^,^2^,^3^,^4^. Deficiencies in transport result in axonal roadblocks of accumulated organelles^4^,^5^,^6^,^7^,^8^,^9^,^10^, potentially contributing to neuronal degeneration.

Modulators of kinesin force generation are unknown, but LIS1, assisted by NudE and NudEL, is implicated in high-load cytoplasmic dynein function. LIS1 is essential for transport of nuclei within neural progenitor cells and migrating neurons in developing mammalian brain^11^. We and others^4^,^12^ have also identified a requirement for LIS1 specifically in axonal transport, and especially involving larger lysosomes/late endosomes^4^. Additional studies also report a broader role for LIS1 in vesicular transport^13^ and less apparent size dependence.

*In vitro* analysis of LIS1 effects on mammalian dynein revealed enhancement and prolongation of the dynein–microtubule interaction during the dynein power stroke^14^. This slower detachment results in better summation of forces generated by multiple cytoplasmic dyneins, and enhanced duration of force output, though peak force was not significantly changed for individual dynein motors. The triple NudE–LIS1–dynein complex also exhibited prolonged stalls under load, and enhanced the duration of force production under multiple motor conditions, allowing improved cargo escape from optical traps^14^. A study of yeast cytoplasmic dynein^15^ proposed a clutch-like role for LIS1, stalling dynein movement along microtubules while ATP hydrolysis persisted (even when load was absent); however, when working with both NudE and LIS1 (ref. 14), mammalian dynein does not stall, and its velocity is unaffected. Despite the evidence in higher eukaryotes for a specific requirement for LIS1 in transport of larger structures^4^,^11^,^12^, direct evidence for dynein force regulation *in vivo* is lacking.

The current study was initiated to directly monitor microtubule motor-generated forces associated with cargo transport in living cells. We used lipid droplets (LDs) as a model system, since their motion is important for metabolism^16^,^17^, and since their basic parameters of transport and protein composition^17^,^18^ are well characterized. Surprisingly, force production was not constant. Rather, the duration of active force production associated with LD transport increased with successive attempts to exit an optical trap. This remarkable adaptive behaviour requires LIS1 and NudE, and can be replicated in a cell-free system, suggesting that cargo-bound factors permit dynamic adjustment of dynein behaviour in response to load.

## Results

### LD escape probability reveals adaptation to load

**Figure 1:**
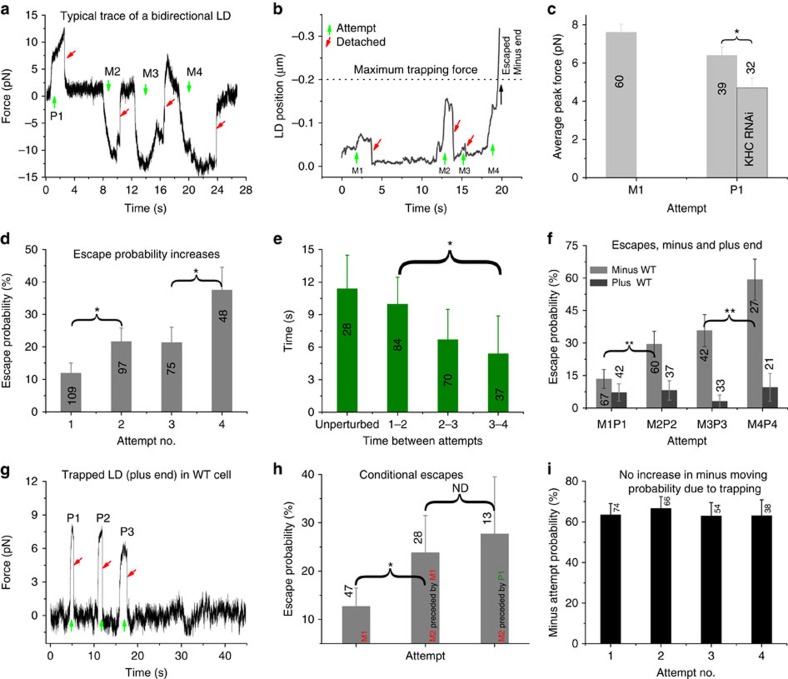
Force adaptation occurs for lipid droplets (LDs) *in vivo* in the minus end direction. (**a**) Typical trace of LD escaping the optical trap. In the figures, ‘M#' and ‘P#' denote direction and escape attempt # of the LD in laser trap, that is, the first escape attempts towards the minus and plus end of the microtubule are denoted by ‘M1' and ‘P1', respectively. Unless mentioned otherwise, the numbers in/above the bars in the figures hereafter are the *N* values for the measurements. (**b**) High resolution bi-directional force trace of a LD showing increased minus-end force persistence and higher force with time. (**c**) Average peak plus end forces decrease due to KHC siRNA treatment (data shown is from 2 different sets of cultures from ∼25 cells and reduced forces were observed in four trials). (**d**) Probability of LDs escaping from the laser trap increases with attempt number. (**e**) Average time between periods of linear motion (Unperturbed) or attempts (when trapped) of LDs in WT cells. (**f**) Percentage of LDs escaping from the trap in the minus- and plus-end directions as a function of attempt number. (**g**) Trace showing lack of adaptation in the plus end moving LD in the trap. (**h**) Minus-end adaptation occurs even when a previous (failed) attempt was in the plus-end direction. (**i**) No change in the probability of a given attempt occurring in the minus end direction as a function of attempt #. (In **d**–**h**, trajectories of 109 LDs were analysed from cells cultured on four different days. At least five cells were analysed in each dish lasting for about an hour. See methods. Overall, force persistence adaptation of LDs was observed in more than 20 different sets of WT cultures.) *P<0.05, **P<0.01, *t*-test Error bars=s.e.m.

*In vivo*, cargos likely experience significant opposition to motion due to the viscoelastic cytosol^19^ and are observed to slow down and subsequently speed up (recoil)^20^ once they escape from an area of increased local opposition. To controllably simulate such a local impediment, we used an unmoving moderate-power optical trap positioned over an individual LD, and examined its escape probability from the trap. In COS1 cells, individual droplets moved in both the microtubule (MT) plus-end and minus-end direction (Fig. 1a, Supplementary Notes 1 and 2). MT staining with Tubulin tracker and expression of EB1-GFP (green fluorescent protein) confirmed that microtubules were oriented radially outward and with plus ends predominantly towards the periphery (Supplementary Fig. 1a–c, Supplementary Movies 1–2). We studied LDs undergoing linear transport toward or away from the cell centre, assumed to be microtubule-associated. The laser trap was positioned over a moving droplet using an automated system, and un-shuttered to capture the LD (see Methods section and Supplementary Note 1). Droplets moving away from the trap centre experience increased resisting force, resulting in either detachment of the LD from the underlying microtubule and return to the trap centre (failed escape attempt), or escape of the LD from the trap (Fig. 1b, Supplementary Fig. 1d and Supplementary Movies 3-12). During the initial movement of a given LD (Escape attempt 1; Supplementary Note 3 for description of ‘attempt'), the effective maximum force in either the plus or minus direction was approximately the same (Fig. 1c, For convenience, we refer to escape attempts in the minus end as ‘M#', where ‘#' is attempt number; similarly, ‘P#' are plus end attempts). A few LD's (∼10%) escaped from the trap, revealing persistent (∼300 nm) generation of forces more than 6 pN. However, surprisingly, the per-attempt escape probability increased with the number of attempts (Fig. 1d). In addition, for those LDs that returned to the trap centre, the time before renewed motility decreased (Fig. 1e). Thus, the LDs exhibited a remarkable form of adaptation, resuming motion after increasingly shorter time intervals (from 10±2.5 to 6±1.8 s; *P*<0.05, *t*-test), and generating more effective persistent forces to allow for marked increases in escape probability.

To better understand adaptation, we examined travel in each direction separately. The escape probability increased only in the minus-end direction (Fig. 1f) and not in the plus end (Fig. 1g, typical plus end track). We also tested whether minus-end adaptation occurred only when LDs were initially moving in the minus end direction and found that escape probability for minus-end directed second attempts increased regardless of whether the preceding attempt was plus end or minus end (Fig. 1h).

### Molecular mechanism underlying adaptation

In *Drosophila* oocytes^1^, LDs are driven by Kinesin-1. We confirmed this to be the case in COS1 cells, using small interfering RNA (siRNA) directed against Kinesin-1. In sufficiently inhibited cells, LD motion effectively ceased, and measurements could not be made in either direction, consistent with previously described transport coupling between plus and minus directions^4^,^21^. In cells in which siRNA inhibition of kinesin-1 was only incomplete (∼60% KHC remaining), plus-end forces were specifically decreased (Supplementary Fig. 1e–h, Supplementary Fig. 2d,e). These results confirmed that COS1 LDs are transported by kinesin-1, and that this motion likely reflects coordinated activity of multiple kinesin motors, both because the maximum forces in this direction appear to show possible intermediate stalls (Supplementary Fig. 2c,d, arrows), because the maximum force is often more than the 4–5 pN force expected for a single kinesin, and because, while droplets still moved, the mean forces driving them decreased with decreasing kinesin.

**Figure 2:**
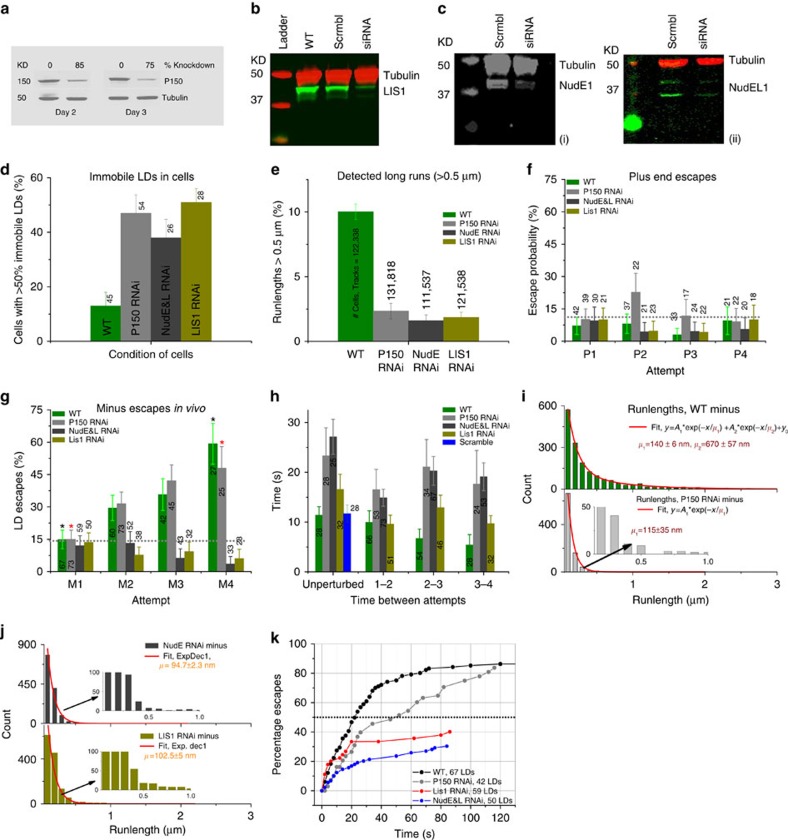
NudE and L/LIS1 contribute to force escape adaptation and dynactin to on-rate adaption. (**a**–**c**) Western blots quantifying the levels of P150, LIS1 and NudE(**i**)& NudEL(**ii**) in control and siRNA treated cells. (**d**) Inside COS1 cells, when LIS1, NudE and L and P150 levels are reduced, there is an increase in immobile LDs. *N* values indicate no. of cells (**e**) In siRNA treated cells, there are fewer long runs (>0.5 microns) compared to the WT. (**f**) Escape percentages in the plus end direction in LIS1, NudE and L and P150 SiRNA treated cells are similar to WT cells. The apparent larger escape probability for P2/P150 is statistically significant but unlikely to be real (Supplementary Note 4) (**g**) Minus end force adaptation is absent in the cells with low levels of LIS1 and NudE and L, but still occurs in the reduced P150 background (**h**) In the decreased P150 background, the time between attempts is longer and does not adapt as it does in the WT. Lack of attempt-frequency adaptation also occurs in the NudE and LIS1 knockdowns. WT data in 2h reflects additional control measurements made simultaneously with the RNAi knockdown measurements, and is thus independent of data presented in Fig 1e. (**i**) Run lengths are decreased in P150 siRNA cells (bottom) relative to the wild type (top). Similarly in NudE/L and LIS1 siRNA cells, LDs have shorter runs(**j**). (**k**)Cumulative percentage of LD population that escaped from the optical trap *in vivo*, as a function of time in WT, P150, LIS1, and NudE and L SiRNA cells. Error bars=s.e.m. In **f** and **g** they are proportional errors. *P<0.05 *t*-test. Reported siRNA phenotypes were observed in at least 5 different days of cultures and at least 20 cells were analysed per set.

Cytoplasmic dynein moves *Drosophila* LDs in the minus-end direction^22^,^23^. In COS1 cells, dynein heavy-chain siRNA caused severe inhibition of total motile LDs. Analysis of effects on plus- versus minus-end-directed movement was not preformed because of disruption in MT organization^24^,^25^. Therefore, we instead examined effects of RNA interference (RNAi) for the two major known dynein cofactor complexes, NudE/LIS1, and dynactin^4^,^14^, implicated, respectively, in force and processivity regulation. We exposed cells to siRNAs to decrease either the major functional subunit of dynactin, p150^Glued^ or LIS1, or simultaneously NudE and NudEL (Fig. 2a–c). In each case, two different sets of siRNA reagents were used, and for each target the functional effects were the same.

LIS1 siRNA treatment resulted in ∼80% or more decrease in LIS1 protein (Fig. 2b). Changes in the cells were not dramatic: LD movement largely persisted, and LDs retained their typical dispersed distribution. Nonetheless, particle tracking analysis revealed that overall LD motion was decreased in both the NudE/L or LIS1 RNAi backgrounds, and mean run-lengths and MSDs decreased considerably (Fig. 2d,e,j and Supplementary Fig. 2l). Interestingly, consistent with a possible inability to overcome obstacles, there was an increase in immobile and clumped LDs (Fig. 2d and Supplementary Fig. 2k).

Clear effects of the LIS1 siRNA treatment emerged under load. Force production was initially similar to the wild type: the first attempt escape probability was the same. This result suggests a similar number of active cargo-associated dyneins, even though LIS1 was reported to contribute to dynein-cargo recruitment in some systems^26^,^27^. Nonetheless, in contrast to control cells, the increasing ability of minus-end droplets to escape the optical trap was abolished (Fig. 2g, Supplementary Movies 13–14), nor did the time interval between attempts decrease (Fig. 2h, green versus gold bar). As in control cells, plus-end escapes did not change (Fig. 2f).

NudE and NudEL siRNA are reported to disrupt vesicular transport more severely than LIS1 siRNA^4^,^12^,^13^,^28^, and indeed, at the highest levels of siRNA treatment (20 nM combined siRNA concentration, comparable to the 20 nM LIS1 siRNA treatment studied), overall motion was severely inhibited, and cells appeared unhealthy (increase in number of rounded and/or detached cells, increased blebbing), consistent with past phenotypic observations for dynein heavy chain knockdown^29^. Instead, we used a more modest treatment (combined 10 nM siRNA), which was still able to significantly decrease NudE/L protein levels (by ∼90%, example western blot (Fig. 2c)), but while preserving overall cellular morphology. Nonetheless, LD motion was decreased (see above). The LD distribution remained relatively unchanged, though there was an increase in immobile LDs (Fig. 2d and Supplementary Fig. 2k). As with LIS1 siRNA, effects under load were clear: there was again wild type like force production on the first attempt, but lack of adaptation in subsequent attempts (Fig. 2g, Supplementary Fig. 2f–i, Supplementary Movies 15–17).

Dynactin is implicated in controlling dynein run lengths^30^,^31^, with effects on processive as well as diffusive movement along MTs^32^. It slightly increases the duration of single-molecule dynein force production^32^, but much less so than NudE–LIS1. We used siRNAs for the major active dynactin subunit, p150^*Glued*^ to reduce polypeptide levels by at least 85% (Fig. 2a). As expected^33^, this decrease reduced overall cellular LD travel, quantified by average RMS displacement (Supplementary Fig. 2i). Restricting our analysis to droplets undergoing linear directed displacements (runs), the proportion of runs longer than 500 nm was reduced fivefold in the p150^*Glued*^ siRNA background relative to wild type (Fig. 2e), similar to the LIS1 and NudE knockdowns, and overall minus-end runs were considerably shorter in the p150^*Glued*^ siRNA background (Fig. 2i) as in the NudE and LIS1 backgrounds (Fig. 2j). Unlike NudE and LIS1, loss of p150^*Glued*^ function did not impair force adaptation. Instead, minus-end escape probability increased just as in the wild-type (Fig. 2g, Supplementary Fig. 2j, see Supplementary Note 4 regarding high value of P2 escape % in P150 KD).

Interestingly, in the p150^*Glued*^ knockdown background, even though mean minus-end forces were unaffected (suggesting unchanged mean number of motors) the interval between attempts was approximately doubled, and did not adapt (decrease) as in the wild type (Fig. 2h). Thus, our analysis identifies a new role for p150^*Glued*^ in dynamically controlling *in vivo* re-attachment-rates of cargos to microtubules, independent of motor number.

In summary, in response to applied load in control COS1 cells, we detect an increase in the probability of minus-end escapes (requiring the NudE/LIS1 system), and a decrease in time between attempts (requiring both NudE and Dynactin). How important are the relative contributions of NudE/LIS1 versus dynactin for overall performance? To quantify this, we looked at T_1/2_, the typical time for half of the droplets to escape from the optical trap. For control cells T_1/2_, was about 22 s (intersection of dotted 50% line with WT population escape curve, Fig. 2k). In the p150^*Glued*^ knock-down background, T_1/2_ increased to approximately 50 s, a factor of ∼2.3. Finally for the NudE or LIS1 backgrounds, we never reached T_1/2_ experimentally (with experiments ending at ∼90 s), so at a minimum T_1/2_ was larger by at least a factor of 4.1, though because T_1/2_ was not reached, the 4.1 factor underestimates the magnitude of effect.

### Force measurements with a high-power trap

**Figure 3:**
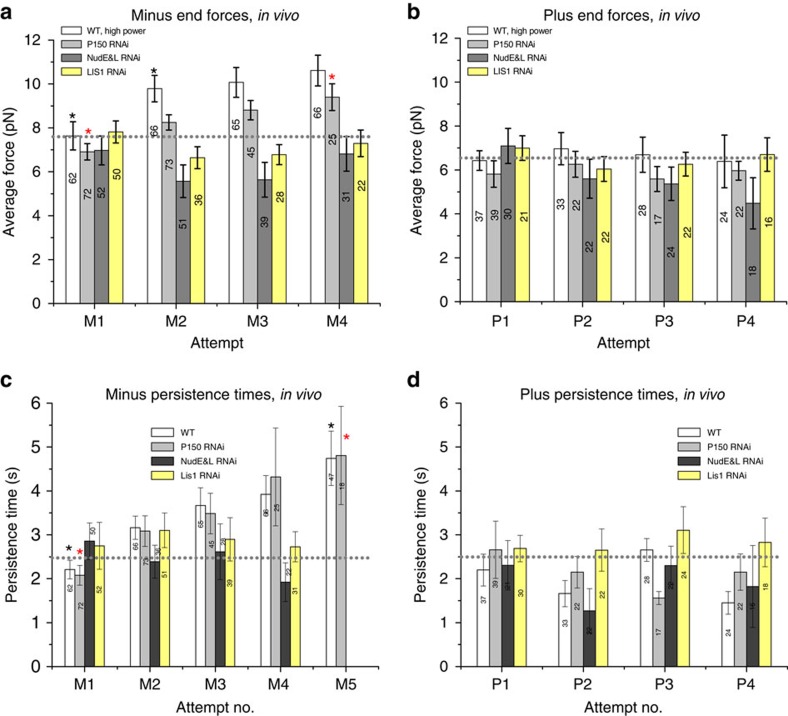
High power measurements in WT cells show LD adaptation reflects increased force persistence (**a**) The average peak forces of all LDs in the minus end direction shows a slight increase for WT and P150siRNA cells but not for LIS1 and NudE&L siRNA treated cells. (**b**) The average peak forces of all LDs in the plus end direction does not change. (**c**,**d**) In WT and P150 siRNA cells, the average persistence time of all attempts increases significantly in the minus end, but not in the plus end, direction. (99 LDs, excluding measurements in Fig. 1, were analysed for WT using high power trap to minimize the escapes). The average persistence time of minus-end LD attempts does not increase in LIS1 or NudE and L siRNA treated cells in any direction. Error bars=s.e.m. *P<0.05, *t*-test.

The increasing escape probability (Fig. 1d, Fig. 2g) could reflect increased maximal force production (for example, due to additional motor recruitment), or alternatively, more persistent attachment to MTs under applied load (that is, ability to walk further/hold on longer under load) if *in vitro* findings^14^ were to apply to *in vivo* function as well. To test these possibilities, we increased the laser power to eliminate almost all escapes, and measured the maximum force that the LDs produced (via changes in the light momentum^34^). Repeated LD movements away from the centre of the trap were associated with an ∼25% increase in maximum minus-end force production in the WT and p150^*Glued*^ siRNA backgrounds; this increase was eliminated in the LIS1 and in the NudE/L siRNA backgrounds (Fig. 3a). Plus-end forces did not change (Fig. 3b).

While the maximum force increase was modest, the increase in the duration of these attempts was larger. Minus-end stall durations increased with escape attempt in both WT and p150^*Glued*^ siRNA backgrounds (Fig. 3c), and minus-end attempts lasting more than 4 s increased from 14% in attempt 1 to 41% by attempt 5 (Supplementary Fig. 3a), which was not observed for plus end movements (Supplementary Fig. 3b). In the LIS1 and NudE and L siRNA backgrounds the initial duration of LD stalls in the stronger trap setting was approximately the same as in the wild-type case (Fig. 3c). Strikingly, however, the duration of stalls in the LIS1 or NudE/L KD cells remained unchanged in subsequent attempts, suggesting these proteins were required for the force adaptation. In the MT plus end direction, in the WT and siRNA backgrounds tested (Fig. 3d), stall durations were unaffected. In conclusion, adaptation occurs only in the minus-end direction, requires NudE and LIS1, and is manifested predominantly with an increased duration of minus-end attempts, though maximal stall forces also increase modestly. In addition, the time between attempts decreases, a process requiring both NudE and dynactin.

### Recapitulation of LD motion *in vitro*

**Figure 4:**
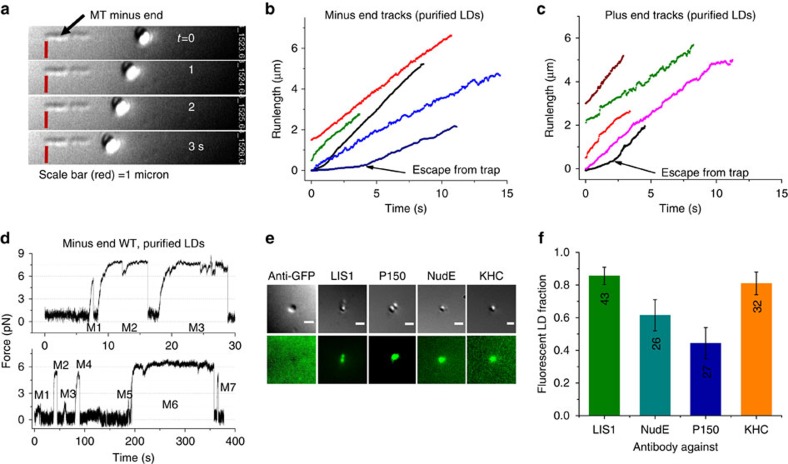
Reconstitution of LD motion and adaptation *in vitro*. (**a**) Purified LD moving towards minus end of polarity marked microtubule (Minus ends are small biotinylated MTs attached with beads prepared as mentioned in Soppina et al.,^57^ (supplement) excepting that beads used are streptavidin coated,150 nm MagCellect from R&D Systems, USA). (**b**,**c**) Typical tracks of purified LDs in the minus and plus end direction respectively. (**d**) Typical minus end trace of purified LD. Note that the lower trace in **d** has a low (atypical) on-rate, but was chosen because it includes a few small attempts (M1 and M3) as well as an extremely long duration event (M6). (**e**) DIC and TIRF images of LDs immuno-stained for LIS1, NudE, P150 and KHC proteins (also see Supplementary Fig. 5). The first panel is the GFP control showing no signal. Control GFP reflects use of a primary anti-GFP antibody (no GFP-labelled proteins present) made in rabbit (same host as the LIS1ab), to confirm that the LIS1 signal was not due to nonspecific binding of anti-LIS1ab. Scale bar=1 μm. (**f**) Fraction of LDs showing the presence of LIS1, NudE, P150 and KHC in the *in vitro* immuno-staining experiments. Error bars=Binomial standard error of proportion.

LIS1 is reported to interact transiently with dynein-cargos in cells^35^,^36^, and outer arm (flagellar) dynein's association with LIS1 is reported to increase under some conditions^37^. Thus, one model to explain LIS1/NudE-dependent adaptation would be new recruitment of cytosolic NudE and LIS1; since NudE can recruit dynein, such recruitment could increase forces by increasing cargo-bound motors, as well as improving how existing cargo-bound motors add forces due to decreased detachment under load. This model was difficult to test *in vivo*. Instead, we tested it in a new *in vitro* system. LDs from COS1 cells were purified using a flotation procedure^38^,^39^ and interactions of the LDs with MTs were examined on addition of ATP. We observed striking, directed LD movement along taxol-stabilized, polarity-marked microtubules (Fig. 4a–d and Supplementary Fig. 4a–g).

These results provide evidence that mammalian COS1 LD transport movement can be reconstituted *in vitro* as observed for some other systems^40^,^41^ . Also important is the implication of a physical association of motors and their cofactors with the purified LDs. To test this, we performed immunocytochemistry and found that the majority of LDs were positive for LIS1, NudE, p150^*Glued*^, and KHC (Fig. 4e,f and Supplementary Fig. 5), even before any application of load from the optical trap. Thus, the key components—LIS1 and NudE—are present and recruitment may be unnecessary.

**Figure 5:**
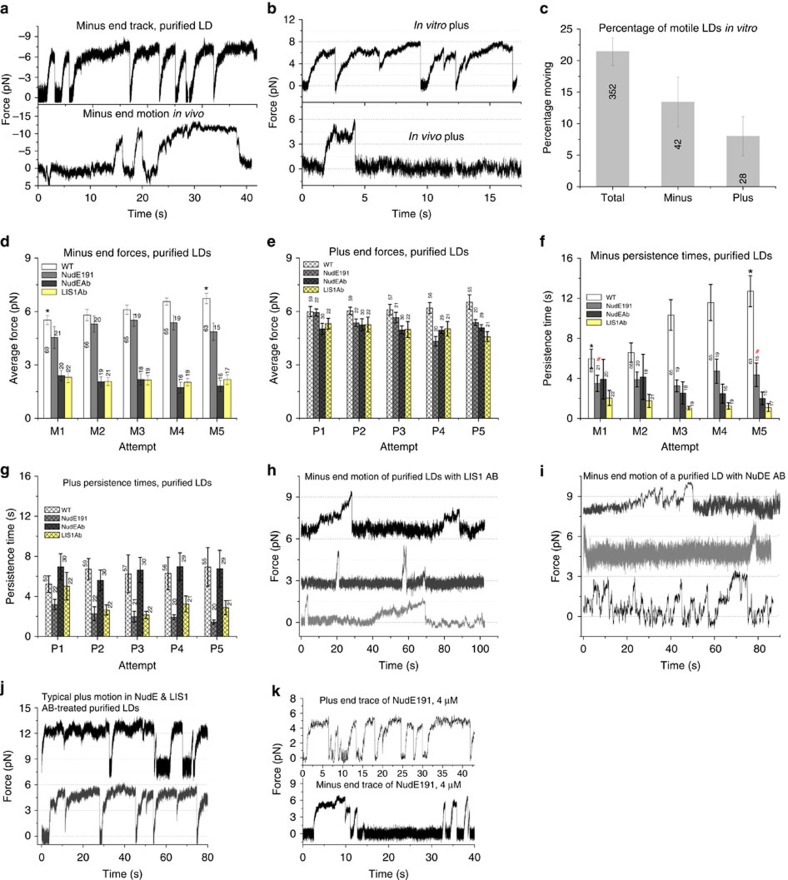
LD motion and adaptation *in vitro*. (**a**,**b**) On-rates and persistence times of motors on purified LDs moving in the minus and plus ends are much higher (top traces) compared to the same *in cells (*bottom traces). (**c**) Motile fraction of purified LDs moving along polarity-marked taxol-stabilized microtubules (∼22%) is slightly larger than that of LDs moving *in vivo* (∼5–10%) at any given instant. (**d**,**e** ) Average forces of WT purified LDs increase slightly in both the directions. Both LIS1 and NudE function blocking antibodies and the NudE fragment reduced the forces. (**f**,**g**) Force persistence times for WT LDs are much higher than *in vivo* in both directions. As *in vivo*, persistence durations adapt and increase in the minus-end direction. Function blocking antibodies to LIS1 and NudE eliminated the adaptation, as did NudE fragment (10-191 aa). (**h**,**i**). Typical traces of Minus end moving LDs illustrating lower forces in the presence of function blocking abs to LIS1 and NudE while the plus end motion (**j**) is unaffected.(**k**)Typical traces of NudE191 treated LDs show relatively short persistence times(compare them to top panels in **a**,**b**). For **d**–**e**, labels in M1 and P1 indicate number of LDs tested. LD motion was observed in more than 10 different purifications. *P<0.05 *t*-test. ^#^Not significantly different. Error bars are s.e.m. for **d**–**g** and proportional errors for **c**.

When the optical trap brought purified LDs into contact with microtubules, we detected a high degree of bidirectional motor activity (Fig. 5a,b, Supplementary Movies 18–20) with ∼ 21% of the LDs binding and moving along MTs (Fig. 5c). Nearly 62% of motile LDs travelled in the minus-end direction, in agreement with our measured probability for minus-end motion *in vivo* (Fig. 1i). The average plus-end force *in vitro* of 6±0.3 pN (Fig. 5e) also matched the *in vivo* value (6.7±0.25 pN, Fig. 3b), though the initial, pre-adaptation minus-end force was slightly lower than the *in vivo* force (5.7±0.27 versus 7.5±0.7 pN) possibly due to the loss of some motors during purification.

We again quantified motion in a high-power trap, to determine whether adaptation was preserved. Strikingly, minus-end stall durations increased dramatically, from ∼6.5 to ∼14 s (Fig. 5f). The initial *in vitro* durations were longer than *in vivo*, but the magnitude of adaptation was the same: *in vitro* the duration increased by a factor of 2.15, and *in vivo* the factor was 2.09 (from ∼2.2 to ∼4.6 s). These results suggest that factors required for force adaptation *in vivo* also co-fractionate with the purified LDs *in vitro* and adaptation is not due to labile MTs (Supplementary Note 5).

Do the already-present droplet-bound LIS1 and NudE contribute to adaptation *in vitro*? To test this, we added a function-blocking anti-LIS1 Ab^4^,^42^ at a concentration of 0.1 mg ml^−1^, and found that there was still minus-end LD motion (Fig. 5h), but that the average force production events were shorter than in controls (Fig. 5d,f), and critically, did not increase with attempt number as they did in the wild type. The antibody also decreased average forces (Fig. 5d), perhaps reflecting general blocking of dynein function. These effects appear specific: plus-end motion was unaffected (Fig. 5e), and the anti-LIS1 antibody did not impair beads moved by purified bovine dynein (lacking LIS1) *in vitro.*

We also perturbed NudE function, first using a NudE function-blocking Ab, previously shown to block NudE activity in cells and to interfere with the NudE–LIS1 interaction^4^,^43^. At an anti-NudE/L Ab concentration of 0.1 mg ml^−1^, minus-end motion could be detected (Fig. 5i), but persistence times no longer increased with attempt number (Fig. 5f). As for the LIS1 ab, average force production was again decreased (Fig. 5d). Thus, both the LIS1 and the NudE/L antibodies blocked adaptation.

As an additional approach we added a NudE N-terminal coiled-coil fragment (10–191) to the purified LDs. This fragment^43^,^44^,^45^ has both dynein and LIS1 binding domains, and with purified LIS1 and dynein can increase the duration of dynein force production in *in vitro* bead assays similar to that occurring due to full-length NudE (Manuscript in preparation). In the presence of this fragment, average minus-end forces were only slightly decreased (Fig. 5d),—but did not adapt—and persistence adaptation was also blocked (Fig. 5f). While it is difficult to know exactly how the 10–191 construct works, we note that it lacks the C-terminal domain, which has been implicated in binding a number of potential cargos, however LD cargo binding by NudE/L is not well understood, so a deeper understanding of this must await further work. Nonetheless, because the 10–191 construct retains both a dynein and Lis1 binding ability, it seems plausible that it may compete with the endogenous NudE/L for dynein and LIS1, so our data is consistent with the possibility that it is acting to sequester LIS1. Combined, the antibody and NudE fragment studies confirmed the utilization of the existing droplet-bound NudE and LIS1, and demonstrated that they are required for the force adaptation observed *in vitro* as well as *in vivo*. Because cytosolic factors are reduced during LD purification (and not added to the buffer), these findings are inconsistent with models of adaptation based on recruitment to the LD of additional NudE, LIS1 and/or dynein.

As dynein can take steps of different sizes^46^, and dynactin can change the dynein step-size distribution^32^, we tested whether step size changes during adaptation *in vitro.* We observed no such change (Supplementary Fig. 4h, and Supplementary Note 6), consistent with a model where adaptation predominantly results from increased interactions of the NudE/LIS1 regulatory system with dynein.

### Modelling to better understand adaptation

**Figure 6:**
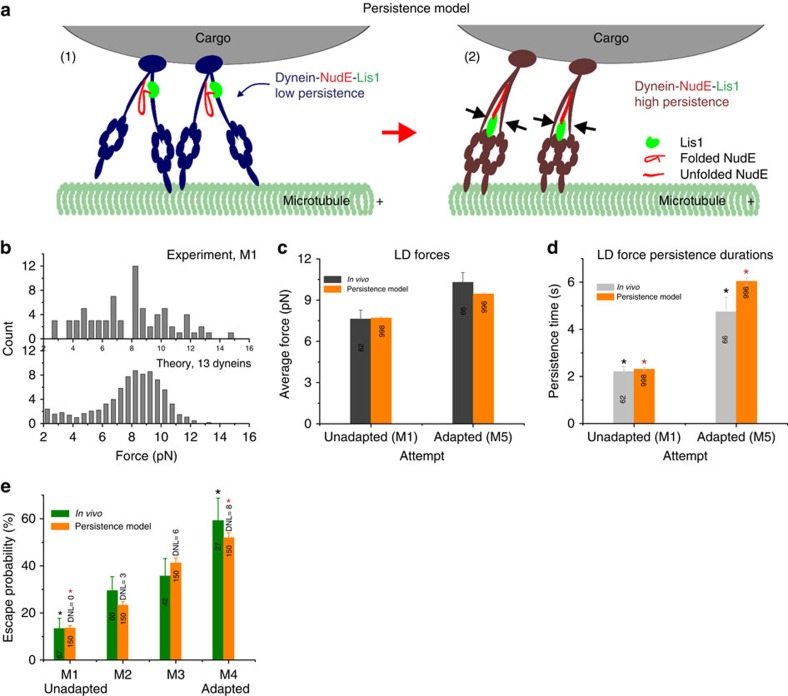
Model. (**a**) Force persistence model to explain the increase in LD escape probability (1) Dynein-NudE-LIS1(DNL) in the unadapted state (2) DNL under high load, after a conformational change in NudE positions LIS1 to interact with the dynein heads allowing it to change their MT detachment dynamics (**b**) Comparison of experiment and theoretical simulations assuming the presence of 13 dynein motors. (**c**,**d**) Average peak force and persistence times from persistence model before and after adaptation agree well with experiment. (**e**) Escape probabilities of trapped LDs from *in vivo* agree well with simulated escapes, using switching/persistence model; number of active DNL complexes assumed as indicated. *P<0.05, *t*-test. Error bars are s.e.m. in **c**,**d** and proportional errors in **e**.

Initially we identified adaptation from the increase in LD escapes from the trap, and our subsequent quantitation demonstrated an increase in mean force, combined with longer-duration attempts. This led to two mechanistic questions: what changes to dynein account for the increased force/duration of attempts, and could these increases quantitatively account for the improved escape probabilities? At the single-molecule level, NudE and LIS1 together slow dynein detachment from microtubules under load *in vitro*^14^, which leads to longer-duration force events, and better additivity of multiple motor forces. Thus, it seemed likely that NudE and LIS1 functioned similarly *in vivo*, reducing the probability of dynein detachment under load (Fig. 6a). Then, since our *in vitro* studies rule out the need for additional recruitment of factors, adaptation would involve increased utilization of already-present NudE and LIS1, potentially controlled via phosphorylation.

The mean LDs forces are higher than those of single vertebrate dyneins^14^,^47^, so suggest the coordinate function of at least five engaged dynein molecules. As this model is based on a reduction in the frequency of detachments of dynein from microtubules, we tested how well this could account for our current observations. Using our previously published Monte Carlo approach^48^ to simulate multiple uncoordinated dynein motors, we could readily theoretically mimic the behaviour of the unadapted cellular minus-end moving LDs in the trap. The simulation parameters (single motor stalling force, velocity, detachment under load), were constrained by previously measured *in vitro* parameters^14^, leaving two inter-related fitting parameters: motor number, and on-rate. We found that a choice of 13 potentially active motors (*N*=13) with a microtubule binding rate (on-rate) of 2.1 s^−1^ matched the un-adapted mean force and duration of attempts (Fig. 6b–d, Supplementary Fig. 3a,c,e–f). Distributions of peak plus end forces and persistence times (Supplementary Fig. 3c and d) fall towards lower end when compared to minus end data. The high number of motors (*N*=13) required to match the *in vivo* forces and times is consistent with a previous *in vivo* measurement^46^. We then modelled adaptation by assuming that load increased the NudE mediated interaction of LIS1 with dynein, and that the effect of NudE–LIS1 interacting with the dynein driving LDs was quantitatively comparable to that which we previously determined experimentally *in vitro*^14^, that is, to increase the dynein's MT binding time under load.

To implement the model, N was fixed but the detachment probability of the motor under load was decreased by ∼50% (ref. 14). When ∼ 61% of the motors used NudE/LIS1 (8 of 13), the model matched the duration of force events well (Fig. 6b–d).

By matching the increase in both mean force and the duration of force production, we thus completely determined the model parameters (*N* value, on-rate, number of dyneins working with NudE/LIS1) for M1 and M5. Then, we used the now-constrained model to test whether the resultant ensemble had correct trap escape probabilities. We first tested the unadapted state: we simulated motion in a fixed-strength optical trap similar to that used in the escape experiments. We found that for *N*=13 dyneins, ∼13 % of the simulated LDs escaped from the fixed trap, in nice agreement with the experimental results (Fig. 6e). We then simulated behaviour of the adapted ensembles, and found that ∼52% of the droplets escaped, again consistent with the experiments (Fig. 6e). Because the model parameters were entirely fixed, these simulations were bona fide tests of the hypothesis that the increase in force duration in the high-force optical trapping experiments are large enough to account for the improved escape-probability.

## Discussion

How cargo force-production is controlled and modulated is poorly understood. Here we identify a novel form of cargo behaviour for motor proteins, the ability to respond to an impediment by upregulating force. By monitoring LDs *in vivo* and in a novel *in vitro* assay we find that the ability to overcome an obstacle (here to escape from a fixed optical trap) improves over time. This response is specific to microtubule minus-end force production, and is manifested in an increase in duration of force production, a slight increase in maximal force, and an increased frequency of force-producing events. Furthermore, the molecular machinery responsible for adaptation is intrinsic to purified LDs. Based on both *in vivo* and *in vitro* analysis, this behaviour uses LIS1 and NudE to control force duration, allowing better ensemble motor function under load. The study therefore provides direct *in vivo* support for such a role for these factors in force regulation, previously proposed on the basis of simpler *in vitro* biochemical and biophysical analysis with purified proteins^14^.

*In vitro*, LIS1 increases the affinity of cytoplasmic dynein for MTs during the transition state: under load, single- dyneins with NudE/LIS1 remain associated with the MT surface for ∼5 times longer than for dynein alone^14^. This allows increased ensemble force output by multiple dyneins, reflecting better summation of their individual contribution due to their longer dwell times. LIS1 inhibition in cultured neurons interfered with larger vesicular transport, consistent with a need for higher persistence, and a role in high load transport *in vivo*. This role for LIS1 is also consistent with its requirement in nuclear migration in developing brain^49^.

The current study further supports for a role for LIS1and Nde1 in high-load transport: we identify a remarkable ability for cargo to adapt to physical resistance. This phenomenon, and the approach taken here, allow us to determine to which specific parameters of cargo transport LIS1, Nde1 and dynactin contribute. LIS1 and Nde1 help control dynein force production *in vivo*. This conclusion is based on the MT minus-end specificity of force adaptation, and the LIS1- and Nde1-dependent increase in both stall forces and in LD force persistence. Furthermore, computational modelling based on LIS1 effects on individual dynein molecules *in vitro* can quantitatively account for the increases in these parameters in our *in vivo* experiments.

Dynactin does not contribute to the major aspect of adaptation involving changes in duration of force production, and its ∼85% decrease does not decrease overall dynein force production. However, it is required for the attempt-frequency adaptation, that is, for the decrease in time between successive binding/motion events. This uncovers a role for dynactin in contributing to the rate at which detached cargos re-bind to microtubules, expanding on a previous report that it helps load cargos on to microtubules plus-ends^50^.

Mechanistically, our data uncover interesting coordination between dynactin and NudE/LIS1, the two major dynein regulatory complexes. Based on a biochemical either/or interaction between the dynein intermediate chain (IC) and either NudE or dynactin^48^, it was hypothesized that dynein functions with only one cofactor at a time. Intriguingly, here both dynactin and the NudE/LIS1 system contribute collectively to ongoing control of cargo run-lengths (Fig. 2e). Although during adaptation, we detect an increasing importance of the NudE/LIS1 complex—without it cargo force durations and mean forces do not increase—nonetheless, NudE/LIS1 clearly contribute to effective normal transport even before adaptation, as indicated by the large increase in time between attempts in the unperturbed case in the NudE knockdown (Fig. 2h), and by the decreased droplet travel distances in both the LIS1 and NudE knockdown backgrounds (Fig. 2j). Conversely, Dynactin continues to contribute to function as adaptation progresses: the attempt-frequency adaption requires both Dynactin and NudE. Thus, our data uncover tight functional integration between NudE/LIS1 and dynactin regulation of dynein, suggesting that binding interactions in addition to the NudE–DIC or Dynactin–DIC interaction must contribute to function of the complex.

Here dynein is more effective than kinesin at overcoming opposition to motion: LD escapes are predominantly in the minus-end direction. While somewhat consistent with a report suggesting dynein is molecularly adapted for this^46^ our data alters this view: in the initial (unadapted) state (attempt 1), both directions are driven by multiple motors, but dynein was not dramatically more effective than kinesin—when mean forces were similar (Fig. 1c) so were the mean durations of sustained force events (Fig. 3c,d; note that duration of P1 is the same as M1), and the probability of escape from the trap was similar (Fig. 2f,g). Instead, it was only after adapting to opposition (requiring NudE and LIS1) that minus-end transport was significantly more effective, suggesting that putative single-molecule properties of unregulated dynein alone are not sufficient to account for these observations. Our *in vitro* data supports this model—on the first attempt, the duration of force in each direction, on polarity-marked microtubules, is the same (Fig. 5f,g). Further, without full-length functional NudE (for example, in the NudE10–191 background *in vitro*), although dynein cargo forces are roughly the same as the kinesin cargo forces (compare Fig. 5d,e), dynein cargo durations are shorter (Fig. 5f,g). Thus, our data suggests that it is the presence of appropriately stoichiometric NudE and LIS1 that result in dynein's superior multiple-motor force production.

It is common to hypothesize that changes in cargo transport result from recruitment or release of factors. However, the *in vitro* studies allow us to eliminate such models here, given that NudE/LIS1 contribute to the process throughout (see above), that they are detected on the majority of LDs *in vitro* (Fig. 4e,f), and that the adaptation occurs with purified LDs where free NudE and LIS1 are absent to be recruited. Thus, while LIS1 contributes to sustained *in vivo* force production, we hypothesize that it does so in a way distinct from what was previously envisioned. Rather than being always active, or recruited in response to load, this *in vitro* data suggest that LIS1 is already present on the cargo and adaptation simply increases the utilization of the NudE/LIS1 system. Our studies thus imply a locally (cargo) triggered mechanism where LIS1 must be spatially juxtaposed with the dynein motor as predicted by (ref. 14), and where NudE contributes to controlling this position. Because the only additive is ATP--and adaptation is statistical in nature (that is, while true on average, the duration of force production/maximal force or the time between attempts for all LDs may not increase (decrease) in a linear manner)—we suspect that the NudE–dynein interaction is under phospho-regulation; confirming this possibility must await further work.

Our findings have multiple implications for dynein-mediated subcellular transport. First, loss of the NudE/LIS1 adaptation system results in significant impairment to cargo force production, and loss of overall ability to overcome obstacles: from the escape curves (Fig. 2k) it seems likely that many cargos with decreased NudE/LIS1 function would not escape the trap. In confined environments such as neurons where cargos experience higher opposition to motion^51^, stuck cargos could cause neuronal blockages. While this adaptation system seems likely to decrease such traffic jams, we do not know the nature of obstacles encountered during transport, nor what is required to overcome them. Nonetheless, other analysis independently suggested the existence of such sub-cellular obstacles^20^. Here the decrease in LD run-lengths in the NudE and LIS1 siRNA cells (Fig. 2j) is consistent with their presence, and with motion that is more sensitive to them. Supporting the challenge of moving large cargos in confined neurons, we previously reported that when the NudE/LIS1 system is impaired, large (but not small) lysosomes cease moving in cultured neurons^4^.

Our data also point to an ongoing role for LIS1 in overall cellular transport. It was suggested that LIS1's cargo interaction is transient^36^, and that it predominantly plays a role in initiating transport. That may be true in some circumstances, but our data suggest that LIS1 plays an ongoing role, since its decrease results in shorter run lengths (Fig. 2j). Since initial forces (in M1) do not decrease in the LIS1/NudE knockdown backgrounds, the observed decreased efficacy of force production likely reflects altered regulation of dynein function, rather than simply changes in the overall number of active dyneins on the cargo, consistent with the observation that *in vitro*, the NudE 10–191 fragment decreases duration of force-producing events, but not maximal (unadapted) force production.

A changing load-dependent LIS1 role allows reconciliation of apparently contradictory observations: in cytosolic backgrounds with low opposition to motion (for example, low amounts of polymerized actin, etc.), LIS1 may interact transiently to promote initiation of transport, but may otherwise be relatively insignificant. However, with higher opposition (due, for example, to increased actin polymerization, or axonal confinement^51^) or in tetrahymena cilia functioning in higher-viscosity environments (where increased recruitment of LIS1 to cilia is reported^37^), LIS1 may remain cargo-bound, and play an ongoing role. Understanding how NudE/LIS1's contribution is dynamically altered remains an exciting challenge for future study.

## Methods

### Cell culture and knockdowns

COS1 cells were grown in DMEM (Invitrogen) supplemented with 10% fetal bovine serum and 1% antibiotic at 37 °C in 5% CO_2_. Before experiments, the cells were synchronized by serum starvation for 24 h followed by release into normal medium for another 24 h. Gene silencing for LIS1, NudE, NudEL and kinesin (KIF5B) was achieved by transfection with commercially available siRNAs from Qiagen. For each knockdown, two sets of siRNAs were used; each set was a pool of two different siRNA duplexes. For LIS1 knockdown the siRNA concentration used was 20 nM and for NudE and NudEL it was 5 nM of each. For KIF5B knockdown the siRNA concentrations used were 50 and 20 nM, respectively. Force measurements were done 48 h post transfection.

The LIS1 siRNAs used were with the following target sequence for set 1 (5′- CACAGCGACTTGCGTTGACAA -3′ and 5′- ATGCGCATGAACACTTTGTTA -3′) and for set 2 (5′- ACGCGTATGGGATTACAAGAA -3′ and 5′- CTCGGGCGCGAGCGCGAGAGAAA -3′).

The NudE and NudEL co-transfection had the target sequences (5′- CAGCGTGCCTTTGGGTGATAA -3′ and 5′- CACGATCATGTCTCTCGAAGA -3′) (NudE) (5′- AAGACTTTGAACAAAGGCTAA -3′ and 5′- CAGTGTTAGAAGATGATTTAA -3′) (NudEL) for set 1 and (5′- CTCCCTAGTGTCTCTGCATAA -3′ and 5′- CGCGCAGACCAAAGCCATTAA -3′) (NudE) (5′- AAGTCAGACTCGGGCCATTAA -3′ and 5′- AAGCTAGAGCATCAATATGCA -3′) (NudEL) for set 2.

The kinesin siRNA used had a target sequence 5′- CTGGCCGAGTGCAACATCAAA -3′. Gene silencing for dynactin1/P150 was achieved with commercially available siRNAs from SCBT. Knockdowns were performed at 1nM using RNAiMAX (Life Technologies). As before, force measurements were carried out 48 h post transfection.

Transfections were carried out using the Hiperfect transfection reagent (Qiagen) following manufacturer's instructions. Cells treated with non-silencing siRNA (Scramble) and transfection mixture without siRNA (Control) were included as controls.

### Immunoblotting

For cell lysates, scraped-off cells were washed with PBS and lysed in ice-cold lysis buffer (25 mM Tris–HCl pH 7.5, 150 mM NaCl, 1% NP40, 1 mM EDTA, 1 mM PMSF, 1 mM Na_3_VO_4_, and 1 × protease inhibitor cocktail from Roche) and the supernatant collected. The proteins in cell lysate were separated in tris-glycine gel. Samples were denatured before loading to the gel using 1:1 (v/v) Laemmli sample buffer and heating at 100 °C for 5 min. The proteins were transferred onto nitrocellulose membrane by wet transfer method and the transferred membrane was blocked with either 5% non-fat milk or bovine serum albumin solution in Tris buffered saline with tween 20 (TBST) for an hour at room temperature. Immunoblotting was done with the respective antibodies and subsequently visualized with infrared detection in Odyssey instrument (Licor). The primary antibodies were diluted in the blocking medium (1:1,000 v/v) and the secondary antibodies were diluted in TBST (1:10,000 v/v). The antibodies were purchased from abcam-56676 (tubulin-α), Santa Cruz Biotechnology, CA, USA (LIS1 sc-15319 , NUDE1 sc-100328), Bethyl (NUDEL1, rabbit-polyclonal ), BD (dynactin/p150 Glued antibody, cat# 610473), Life Tech (Goat anti-mouse IgG 680) and Licor( goat anti-rabbit 800 nm).

### Force measurements in cells

LD positions in the laser trap were measured with high resolution using position sensitive detector (PSD) and cross verified with analysis of differential interference contrast (DIC) images using template matching or autocorrelation of LD intensity profile. Along with real time template matching, a linear *xy* stage and piezo *xy* mirror were employed to improve the accuracy of trap positioning on the moving LD (See Supplementary Note 1 for details of the setup). Typically, both failed and successful escape attempts could be observed in the video as LDs that failed to escape would fall back to the trap centre with high velocity. However, only high resolution PSD data (2 kHz) was used to quantify parameters of the escape attempts (position traces carried very high slope due to rapid fall back to trap centre) with better accuracy. During force measurements double trapping of LDs was quite common, and care was taken to analyse only those LDs whose motion was uninterrupted by other organelles. The ideal region of the cell for measurements is halfway between periphery and nucleus with an additional condition that there is a linear inward and outward flow of organelle traffic to rule out the ambiguity in the direction of MTs. Note that success rate of trapping linearly moving LDs that last for 5 unperturbed attempts in the cell is very low and typically we scored a maximum of 6–8 clean LD tracks in 1 h. Errors in escaped fractions in each attempt (*f*) were estimated with 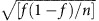 for *n* droplets that made the escape attempt.

### Force calibration, Method-1

Using the standard size silica beads in sucrose solution and QPD.

Force calibration was carried out as reported elsewhere^1^. To describe it briefly, a power spectrum was recorded for the silica beads of known diameter (300–1,000 nm) immersed in sucrose solution that has matching refractive index of cytoplasm (*n*=1.365)^1^. The trap stiffness is sensitive to the refractive index mismatch between the medium and the trapped object. We used 20% sucrose solution to immerse the standard silica beads of known diameters as the mismatch in refractive indices for this combination matches with that of LDs and cytoplasm. The trap stiffness for each bead diameter for a fixed laser power was calculated using the rollover frequency of the power spectrum and viscosity of the sucrose solution. DIC images of the silica beads of known diameters in 20% sucrose solution were used as standards to estimate the diameter of the LDs inside the cells. Sizes of the LDs were estimated by comparing the DIC images of the Silica beads of known diameter (300–1,000 nm) in index matched solution (Supplementary Fig. 6a–c). We fitted sum of two Gaussians (dark side and light side of LDs in DIC image, Supplementary Fig. 6a) to the averaged intensity profile of the still LD in 10 successive images and estimated the size as peak to peak distance plus the standard deviations. Size distribution of trapped LDs in our measurements was in the range of 400 to 760 nm. At 500 mA laser current, the calibrated trap stiffness for such LDs at 100 nm from the trap centre, depending on the size, varied from 5.2 to 9.3 pN (Supplementary Fig. 6c). To generate the tracks of the LD motion in the trap, video tracking was carried out using custom written LabVIEW routines and LVcor software^52^.

### Force calibration, Method-2. Detection of momentum transfer using PSD

This method reported by us and others in refs 53, 54, 55 is independent of the size and shape of the cargo and is thus advantageous in cells. However, it needs high numerical aperture condenser and a careful alignment of the trap to ensure that no refracted light from the trapped object is lost. Here the force experienced by the cargo is directly measured as F=**m**·*V*. Where **m** is the calibration factor (**m**=**k**_trap_ × **β**) and *V* is the voltage signal from the PSD. We estimated the **k**_trap_ (pN nm^−1^) and **β** (nm volt^−1^) by oscillating the trap at 32 Hz and measuring the power spectrum^55^. Following the procedure described in refs 53, 54 and with some additional temperature corrections^55^, our setup's **m** was experimentally determined to be 33 (Supplementary Fig. 6d). Using the momentum transfer method with this calibration factor, we estimated the stall force of purified single molecule full length kinesin-1 from drosophila as ∼4.6 pN (Supplementary Fig. 6e), consistent with other measurements on the scope on the same protein using the QPD/bead position detection approach. The measured ∼4.6 pN stall force is also consistent with previous QPD measurements for K560 where the average force is found to be ∼4.8 pN (ref. 56). Thus, both calibration methods are in agreement.

### Sample preparation for force measurements in cells

During the force measurement experiments we developed a chamber to prepare the cells for imaging up to 2 h without affecting the cell morphology or organelle traffic. To improve cell adhesion the pre-cleaned cover slips were coated with polylysine (P8920 Sigma, 0.7 ml in 250 ml of denatured ethanol) for 12 min and dried for 8 min at 100 °C. Cells were attached to coverslips at least 5 h before the measurement. To hold the cell culture medium a rectangular chamber measuring ∼ 20 × 40 mm^2^ was constructed using microscope glass slide (1 × 25 × 75 mm^3^), cover slip (22 × 40 × 0.17 mm^3^) and double sided adhesive tape (50 microns thickness) . The chamber was filled with warm culture medium from the dish used for culturing the cells (about 100 μl) and the coverslip with attached cells was transferred on to microscope glass slide in such a way that cells were always in contact with the medium (Supplementary Fig. 6g). The residual medium on the top surface of the coverslip, with no cells attached to it, was cleaned up to improve the clarity of DIC images.

### LD purification

LDs were purified using a modified protocol reported in ref 40. COS1 cells were cultured as mentioned before and cells from eight 100 mm dishes (80% confluency) were used per isolation. Cells were washed twice with ice cold PBS and collected by gentle scraping in 0.5 ml of 1.4 M MEPS (35 mM PIPES, pH 7.2, 5 mM EGTA, 5 mM MgSO_4_ and 1.4 M sucrose) supplemented with 2x protease inhibitor cocktail (Roche Mini-Complete tablets). Nitrogen cavitation (800 psi for 15 min) was used for lysing. Lysate was passed through a 20 gauge needle 5 times before centrifuging for 10 min at 1,800 *g* to clear large cellular debris. The supernatant (1.4 M sucrose buffer) was then layered at the bottom of a sucrose gradient with equal volumes of 1.0 M, 0.5 M, then 0 M sucrose buffers (molarities indicate sucrose in MEPS) containing, 1x protease inhibitor, 0.5 mM ATP and 1 mM DTT. Lysates were centrifuged at 170,000*g* for 1.5 h at 4 °C. After centrifugation, the top layer was collected and supplemented with 2.5 M MEPS to a final concentration of 0.5 M MEPS and snap frozen with liquid nitrogen(15 ul aliquots) to store them at −80 °C for further use.

### Polarity labelled MTs for *In vitro* LD forces

Sample chamber was constructed to hold about 20 microliter of solution using glass slide, coverslip and adhesive tape (see sample preparation Method above for specifications). To identify minus end moving purified LDs polarity labelled microtubules were constructed by elongating small axonemes (1–5 microns) purified from Sea Urchins or biotinylated MT seeds using a slightly modified method reported in ref. 57. In this study, MagCellect streptavidin conjugated beads (∼150 nm) were used instead of 350 nm avidin magnetic beads used in ref. 57 to label the biotinylated MT seeds that distinguished minus ends from plus. When used at 1:100 in the MT buffer (80 mM PIPES, pH 7.2, 1 mM EGTA, 1 mM MgSO_4_ and 1 mM GTP), MagCellect beads readily bound to the seed portion of the elongated MTs on the coverslip without any magnet ( in ref. 57 a magnet was used to enhance effective binding of magnetic beads to biotinylated seeds). The beads visible in DIC served as markers for minus end (Fig. 4a). Alternatively, axonemes in MT buffer were attached to surface of clean coverslips (of sample chamber) by incubating them for 5 min at room temperature (RT). Unattached axonemes were removed by washing the chamber with 30ul of MT buffer supplemented with 1mg ml^−1^ casein. Elongation of attached axonemes was carried out using purified tubulin (0.5 mg ml^−1^, Obtained from porcine brain, Prof Les Wilson's Lab) in MT buffer at 37 °C for 25 min. In both cases the elongated MTs were stabilized by flowing MT buffer containing 20 micro molar Taxol (Sigma–Aldrich).

For force measurements, one 15 ul aliquot of LDs from -80C was quickly thawed and supplemented with 1 mM ATP and 100 mM each of Glucose and oxygen scavenging system before flowing it into the measurement chamber attached with static MTs. For each thawed aliquot, force measurements were carried out for ∼30 min at RT using the optical trap and momentum change method that was also used for *in vivo* force measurements. Laser current used for measurements was adjusted to have the trap stiffness of ∼ 6 pN at 100 nm from the centre. Purified truncated kinesin(K-560) motility was carried out as reported earlier^58^.

### Function blocking using antibodies and fragment NudE

Quickly thawed LDs from −80 °C were incubated with 2.5 mg ml^−1^ casein buffer for 3 min to block the surface. LDs were then incubated with function blocking Abs at desired concentration (LIS1 Ab and NudE Ab, 0.1 mg ml^−1^, 1mM ATP) at room temperature for 8 min before testing their motility (supplemented with 100 mM each of Glucose and Oxygen scavenger) using the *in vitro* measurement chamber. The effect of NudE10–191 on LD motility was tested at 4 micro molar following the same incubation and measurement procedures used for NudE Ab.

Function blocking antibodies used against LIS1 and NudE are same as reported in refs 4, 43 respectively. The fragment of NudE amino acid sequence 10–191(NudE10–191) was purified using the method described in ref. 35, under DNA cloning and protein purification methods.

### Microtubule and EB1-GFP Imaging

The imaging was carried out using custom built objective based total internal reflection fluorescence microscope (TIRFM, 1.49 NA, × 100 oil objective, Nikon). Living COS1 cells were attached to the coverslips using the same protocol adopted for force measurements. Before flipping the coverslips with attached cells on to the sample chamber for TIRF imaging, the cells were stained with the vital Tubulin tracker dye (∼5 nM) for 5 min at 37 °C. Recombinant EB1-GFP plasmid (human EB1 in pEGFP-N1, Addgene plasmid 39299) was amplified and purified using XtraMaxi (Nucleobond, 740414.10) from transformed E.coli (Dh5alpha) before transfection into COS1 cells to transiently express the protein and visualize plus ends. After 48 h of EB-GFP transfection, using lipofectamine as in siRNA protocol, nearly 50% of the cells were found to express detectable green fluorescence. The images were acquired, every 2 s, with QuantEM 512SC EMCCD camera (Photometrics, Inc.) attached to TIRFM. Excitation light (488 nm, continuous wave Ti-Sapphire laser, Coherent, Inc.) was kept low to avoid photo toxicity and bleaching. Combination of high NA objective and an automated translation stage (Thorlabs, Inc.) allowed us to tune the depth of excitation, by precise control of the incident angle of excitation laser, in semi-TIRF mode.

### Immunofluorescence of LDs

LDs were incubated with primary antibody at 1:100 dilution overnight on a rotator at 4C in 0 M MEPS buffer containing 1 mg ml^−1^ casein. Unless specified all antibodies used were same as those used for immunoblotting. LDs were then incubated with secondary antibody at a 1:100 dilution in 0 M MEPS buffer for 4 h. Secondary antibodies were Alexa 488 goat anti-mouse or anti-rabbit from Life Technologies. LDs incubated with no primary antibody (only secondary antibody added) were used as controls. For LIS1 detection, as a second control, a rabbit polyclonal anti-green fluorescent protein (GFP) antibody was used to confirm that the signal was not due to nonspecific binding of primary antibodies (GFP is not detected on the LDs). To measure fluorescence signal from the LDs, a sample chamber was constructed using polylysine coated glass coverslip (0.17 mm, Fisher Scientific) and double sided adhesive tape similar to *in vitro* motility assay^59^. Solution containing LDs and antibodies was supplemented with additional casein (2.5 mg ml^−1^) immediately before flowing into the sample chamber. After 10 min of incubation at RT (inside a black box) enough LDs were found attached to the polylysine coated coverslips of the chamber. After incubation, the chamber was washed with 30 micro liter of imaging buffer (80 mM PIPES, pH 7.2, 1 mM EGTA, 1 mM MgSO_4_, and 0.1 M Glucose and Oxygen scavenging system) to reduce the signal from the bulk. Fluorescence imaging was carried out using TIRFM suitable for single molecule imaging (same set up used for EB1-GFP imaging).

### Theoretical model for minus end force adaptation

For the current analysis, we fixed the following parameters based on the *in vitro* data for single motor dynein and single dynein–NudE–LIS1 (DNL) complex^14^. Load-dependent motor stepping rates (forward and backward) and detachment kinetics were set as reported earlier^14^,^48^.

Briefly, we used the following theoretical relations.

Sub-linear force-velocity dependence^48^,^60^;


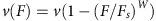


Forward stepping rate,


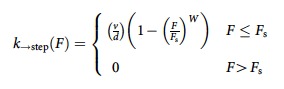


Backward stepping rate,


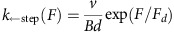


Exponential detachment kinetics below stall^61^


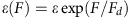


Advancement of the moving cargo inside the trap between any time interval *t* and *t*+Δ*t* is


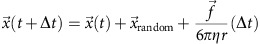


Force on the cargo due to multiple dynein motors was calculated as


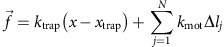


In the above equations, *F*—force on the motor head, *F*_s_—stall force of the motor (1.5 pN), *F*_d_-detachment force (1.2pN), *v—*velocity of the motor (1,000 nm s^−1^ ), *d*—step size of the motor(8 nm), *k*_trap_—laser trap stiffness(0.07 pN nm^−1^ ), *k*_mot_—stiffness of the motor(0.32 pN nm^−1^), Δ*l*_*j*_ is extension of the walking motor *j* beyond its rest length *l* (50 nm), *r*—radius of the cargo (250 nm), 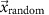
—Brownian displacement, Δ*t*—time step, *B*—Back stepping factor(40), *η*—Viscosity of cytoplasm(∼10 × water, 0.01 N s m^−2^).

In simulations, the number of dynein motors (*N*) required to match the force and persistence time of first minus attempt, M1 was found to be 13. Data points for bead and motor positions were generated for each Δ*t* of 10^−6^ s and maximum duration for the simulations was fixed at 100 s. Parameters optimized to match forces and persistence times and escape probabilities of M1 are; motor binding rate to MT, *π*=2.1 s^−1^, off-rate below stall *ɛ*=1 s^−1^, off-rate above stall *φ*=10 s^−1^ and *N*=13. To simulate LD tracks of M1 to M5 using the force persistence model, N was kept fixed at 13 and number of dyneins that function as DNL complexes was increased (DNL=0, 3, 6, 8 and 13). As reported earlier, whenever a dynein was replaced by a DNL complex it was assumed to have higher force persistence/lower microtubule unbinding rate compared to dynein alone^14^ and hence ɛ and φ were decreased to 0.6 and 6.5 s^−1^, respectively, for that DNL complex. In other words, DNL=3 will have three dyneins in complexes with NudE–LIS1 and nine dyneins functioning independently. To realize this in the simulation, we assumed lower off-rates for the motors working with NudE and LIS1 as they hold on to MT longer than dyneins. Simulations matched the experimental data of M5 for DNL=8(Fig. 6c–e, Supplementary Fig. 3e,f). To match forces and persistence times one thousand tracks (*n*=1,000) were simulated for each attempt.

Escape percentages were estimated by considering the *k*_trap_ to be maximum at 200 nm and by increasing number of DNL complexes (*n*=150 tracks). Trap stiffness was set to 10.5 pN at 200 nm which is close to the experimental value. In the simulations, whenever the bead position crossed 200 nm it was scored as an escape. With this *k*_trap_ and rest of the parameters same as in M1, the escape probability was ∼13% and went up to 52% for *N*=8 DNLs (Fig. 6e) which is in agreement with the experimental value.

### Data availability statement

All relevant data are available from the authors on request.

## Additional information

**How to cite this article:** Reddy, B. J. N. *et al*. Load-induced enhancement of Dynein force production by LIS1-NudE *in vivo* and *in vitro*. *Nat. Commun.* 7:12259 doi: 10.1038/ncomms12259 (2016).

## Supplementary Material

Supplementary Information Supplementary Figures 1-6, Supplementary Notes 1-6, Supplementary References.

Supplementary Movie 1 Typical movies showing the LDs inside COS1 cells escaping from the optical trap towards center after force adaptation. Scale bar = 1 micron.

Supplementary Movie 2 Typical movies showing the LDs inside COS1 cells escaping from the optical trap towards center after force adaptation. Scale bar = 1 micron.

Supplementary Movie 3 Typical movies showing the LDs inside COS1 cells escaping from the optical trap towards center after force adaptation. Scale bar = 1 micron.

Supplementary Movie 4 Typical movies showing the LDs inside COS1 cells escaping from the optical trap towards center after force adaptation. Scale bar = 1 micron.

Supplementary Movie 5 Typical movies showing the LDs inside COS1 cells escaping from the optical trap towards center after force adaptation. Scale bar = 1 micron.

Supplementary Movie 6 Typical movies showing the LDs inside COS1 cells escaping from the optical trap towards center after force adaptation. Scale bar = 1 micron.

Supplementary Movie 7 Typical movies showing the LDs inside COS1 cells escaping from the optical trap towards center after force adaptation. Scale bar = 1 micron.

Supplementary Movie 8 Typical movies showing the LDs inside COS1 cells escaping from the optical trap towards center after force adaptation. Scale bar = 1 micron.

Supplementary Movie 9 Typical movies showing the LDs inside COS1 cells escaping from the optical trap towards center after force adaptation. Scale bar = 1 micron.

Supplementary Movie 10 Typical movies showing the LDs inside COS1 cells escaping from the optical trap towards center after force adaptation. Scale bar = 1 micron.

Supplementary Movie 11 Time lapse movie of microtubules plus ends imaged with TIRFM in EB1-GFP expressed COS1 cells showing majority of Plus ends away from cell center. Scale bar = 3 microns.

Supplementary Movie 12 Time lapse movie of microtubules in COS1 cells imaged with TIRFM using Tubulin tracker dye show negligible motion. Scale bar = 5 microns.

Supplementary Movie 13 Typical videos of LDs in LIS1 knock down cells showing no adaptation of LDs in escaping the optical trap. Scale bar = 1 micron.

Supplementary Movie 14 Typical videos of LDs in LIS1 knock down cells showing no adaptation of LDs in escaping the optical trap. Scale bar = 1 micron.

Supplementary Movie 15 Typical videos of LDs in NudE & NudEL knock down showing the LDs unable to escape from the optical trap (~ at the center of field of view). Scale bar = 1 micron.

Supplementary Movie 16 Typical videos of LDs in NudE & NudEL knock down showing the LDs unable to escape from the optical trap (~ at the center of field of view). Scale bar = 1 micron.

Supplementary Movie 17 Typical videos of LDs in NudE & NudEL knock down showing the LDs unable to escape from the optical trap (~ at the center of field of view). Scale bar = 1 micron.

Supplementary Movie 18 Motion of purified LD along polarity labeled microtubule showing very long force persistence and quick rebinding (high on-rate). Scale bar = 1 micron.

Supplementary Movie 19 Motion of purified LD along polarity labeled microtubule showing very long force persistence and quick rebinding (high on-rate). Scale bar = 1 micron.

Supplementary Movie 20 Motion of purified LD along polarity labeled microtubule showing very long force persistence and quick rebinding (high on-rate). Scale bar = 1 micron.
