# Supplementary material for: Load-induced enhancement of Dynein force production by LIS1–NudE in vivo and in vitro
Source: Nat Commun. 2016 Aug 4;7:12259. doi: 10.1038/ncomms12259 (PMC4976208; doi:10.1038/ncomms12259)
Supplement: Supplementary Information — Supplementary Figures 1-6, Supplementary Notes 1-6, Supplementary References. [file ncomms12259-s1.pdf]

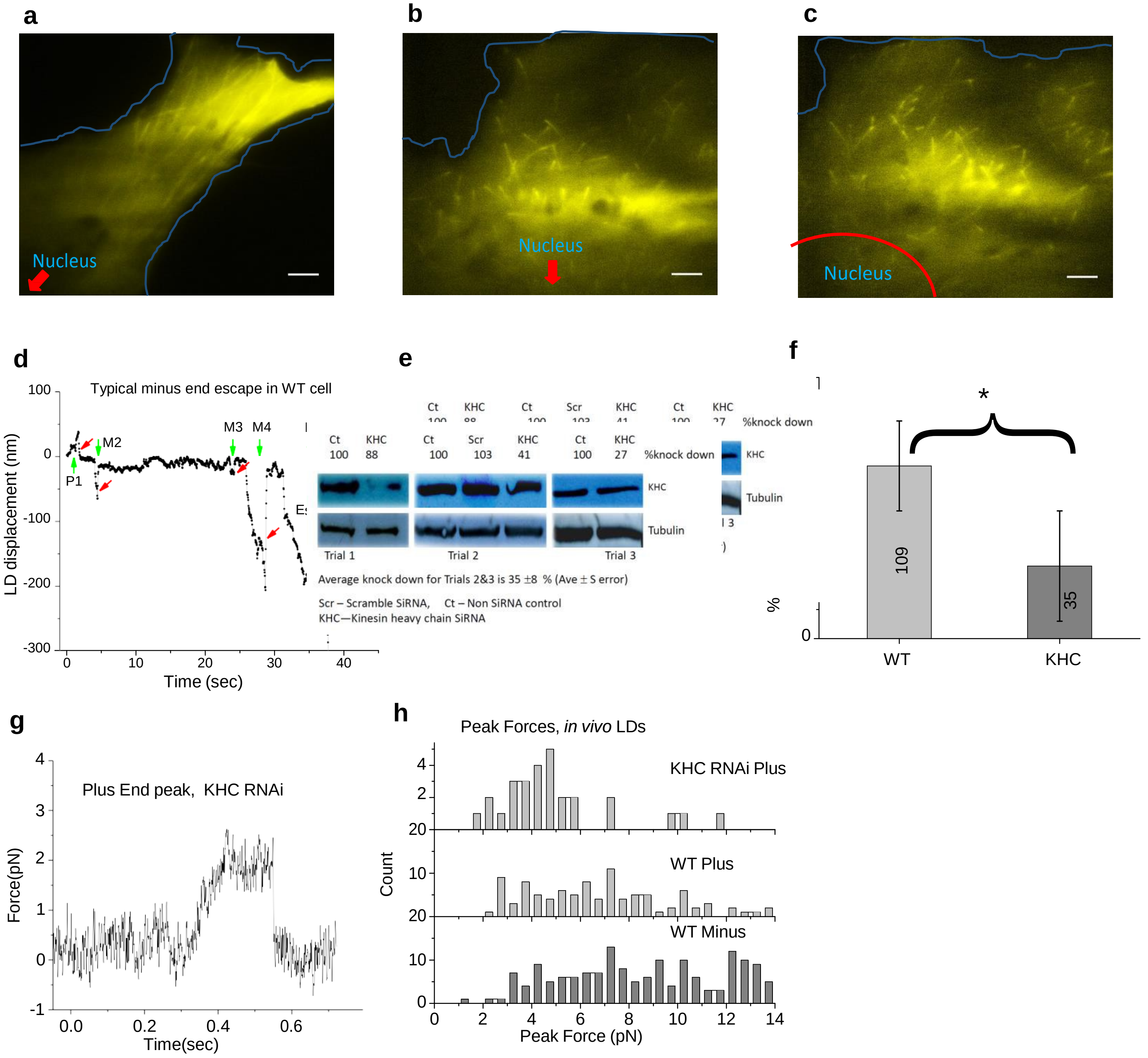

Supplementary Figure 1. Western blots to quantify protein levels and transport related data in the KHC siRNA treated cells**.** (**a-c**) Images of MT plus ends visualized in TIRF Microscopy with GFP tagged microtubule end binding protein (EB1-GFP) in COS1 cells. TIRF images reveal that majority of plus ends are oriented in radially outward direction, scale bar =3 microns (**d**) Additional example of minus end escape in WT COS1 cells (**e**) Western blots of KHC siRNA treated cells. (**f**) Initial escape probability of LDs decrease due to KHC siRNA treatment (Attempt#1). (**g**)Typical small LD force event in KHC RNAi cells. (**h**) Distribution of observed peak forces in the KHC siRNA and Wild Type backgrounds.

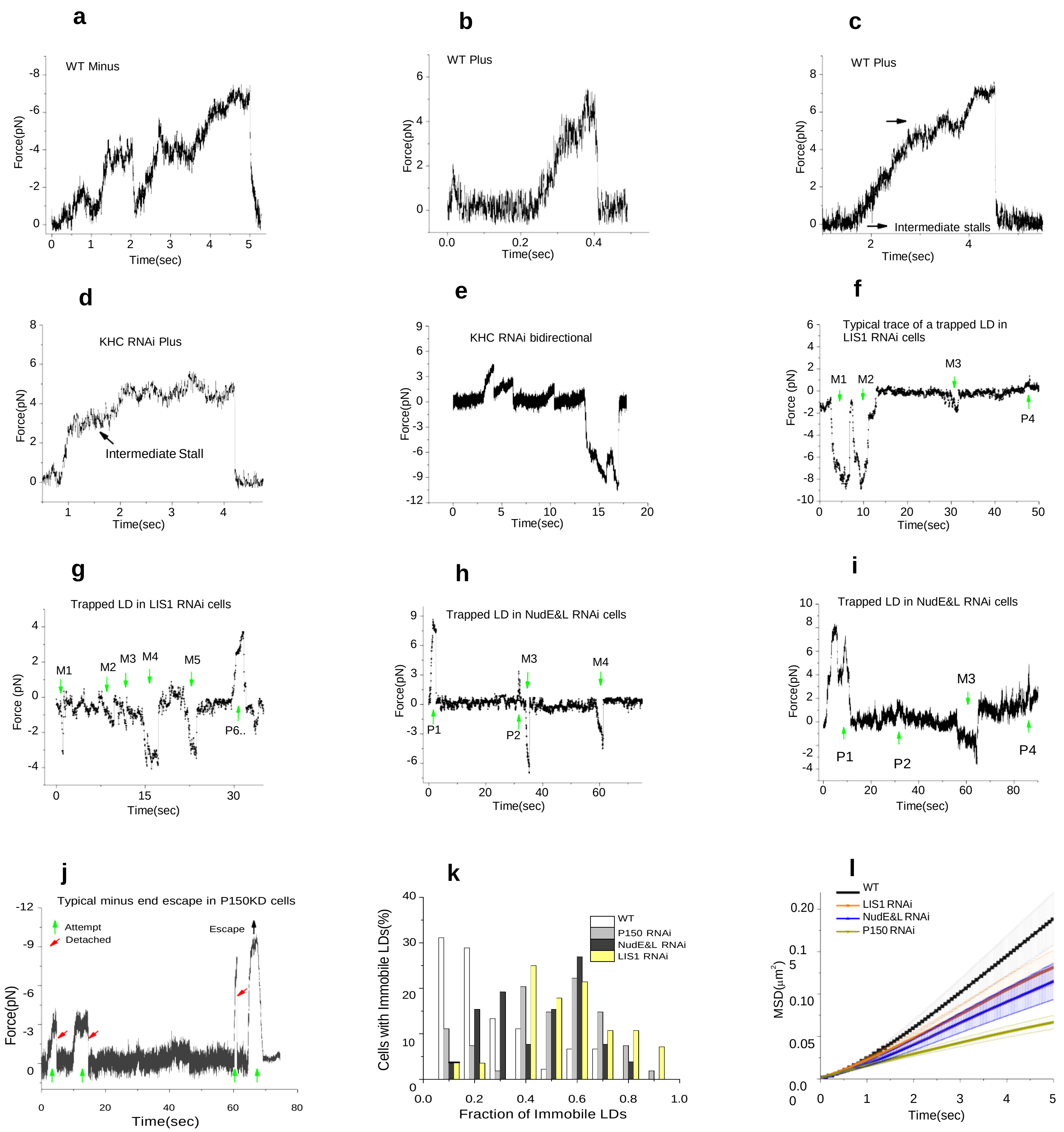

Supplementary Figure 2. Additional example traces and characterization of LD motion. (**a**). Minus end (**b**) &(**c**) Plus end motion in WT (control) cells. All example traces recorded using momentum transfer method for force determination, at 2/4 kHz. (**d**) Plus-end motion in KHC knockdowns. (**e**)An example showing low force plus-end attempts followed by high force minus attempt in a KHC RNAi cell. (**f**) & (**g**) Typical long-time traces of LD motion in the LIS1 SiRNA cells and (**h**) & (**i**) in NudE/L siRNA cells. Visual inspection (evident in these traces) suggests that typical LD force production in the NudE/LIS1 knock-down backgrounds decreases after attempt 1, and overall appears less robust in these traces, compared to motion in controls (in Fig.1). (**j**) Typical minus end escape in P150 RNAi cells. (**k**)Probability distributions of immobile LDs in WT and siRNA treated cells (**l**) Mean square displacements of lipid droplet motion in the COS1 cells. MSDs of LDs from the LIS1, NudE&L and dynactin RNAi cells are lower than that of WT cells (21 tracks from 7 cells in each condition were analyzed for MSD).

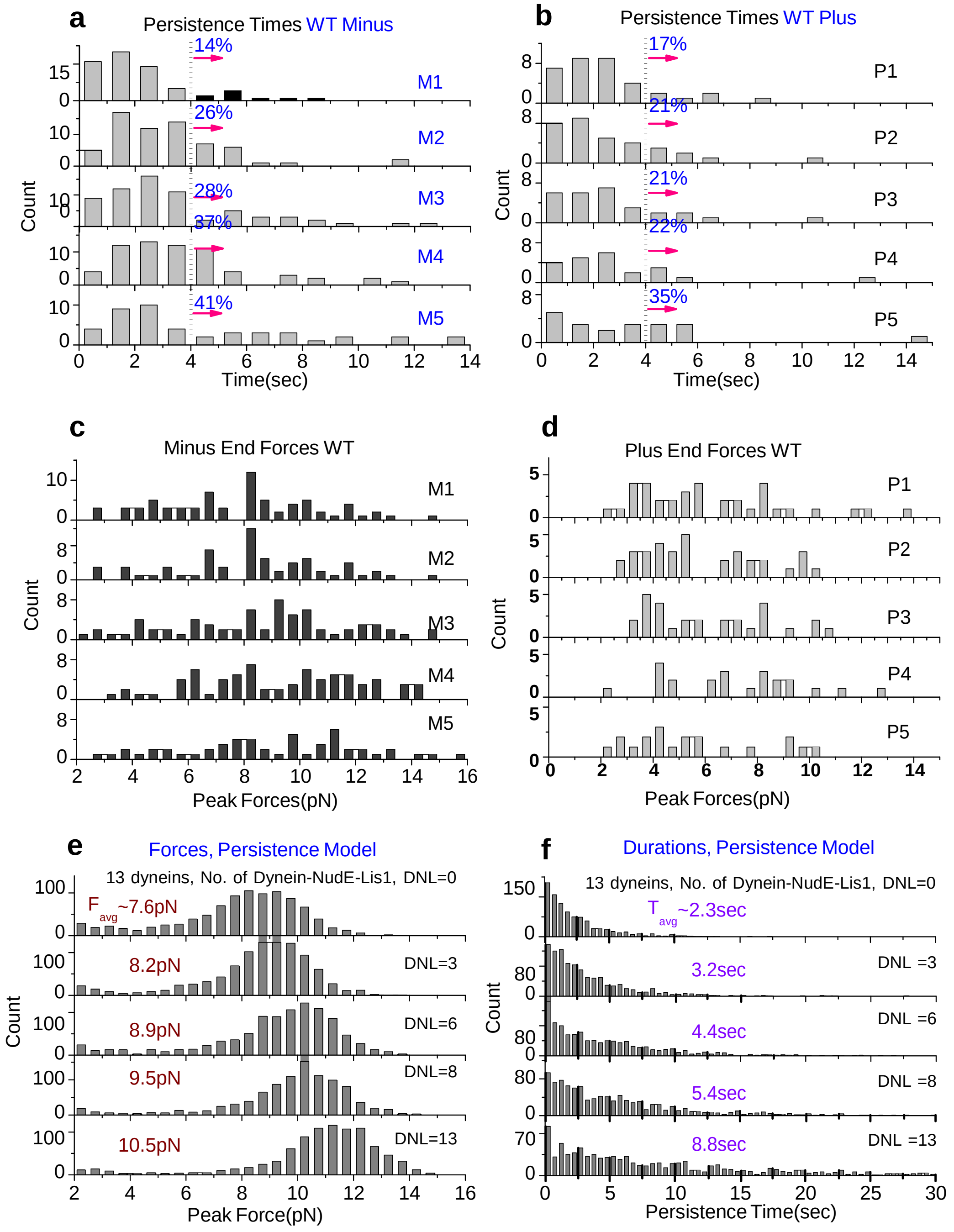

Supplementary Figure 3. Histograms of overall Persistence times and peak forces experiment & theory and Clustering Model **(a**&**b)** Distribution of persistence times in the minus and plus direction respectively with increasing number of attempts. (**c&d**) Distribution of peak forces in the Minus end and Plus end direction respectively with increasing number of attempts. **(e) &(f)** Forces and persistence times of multiple dynein motor cargos from Monte Carlo simulations assuming increased use of high persistence Dynein-NudE-LIS1 complexes under load .

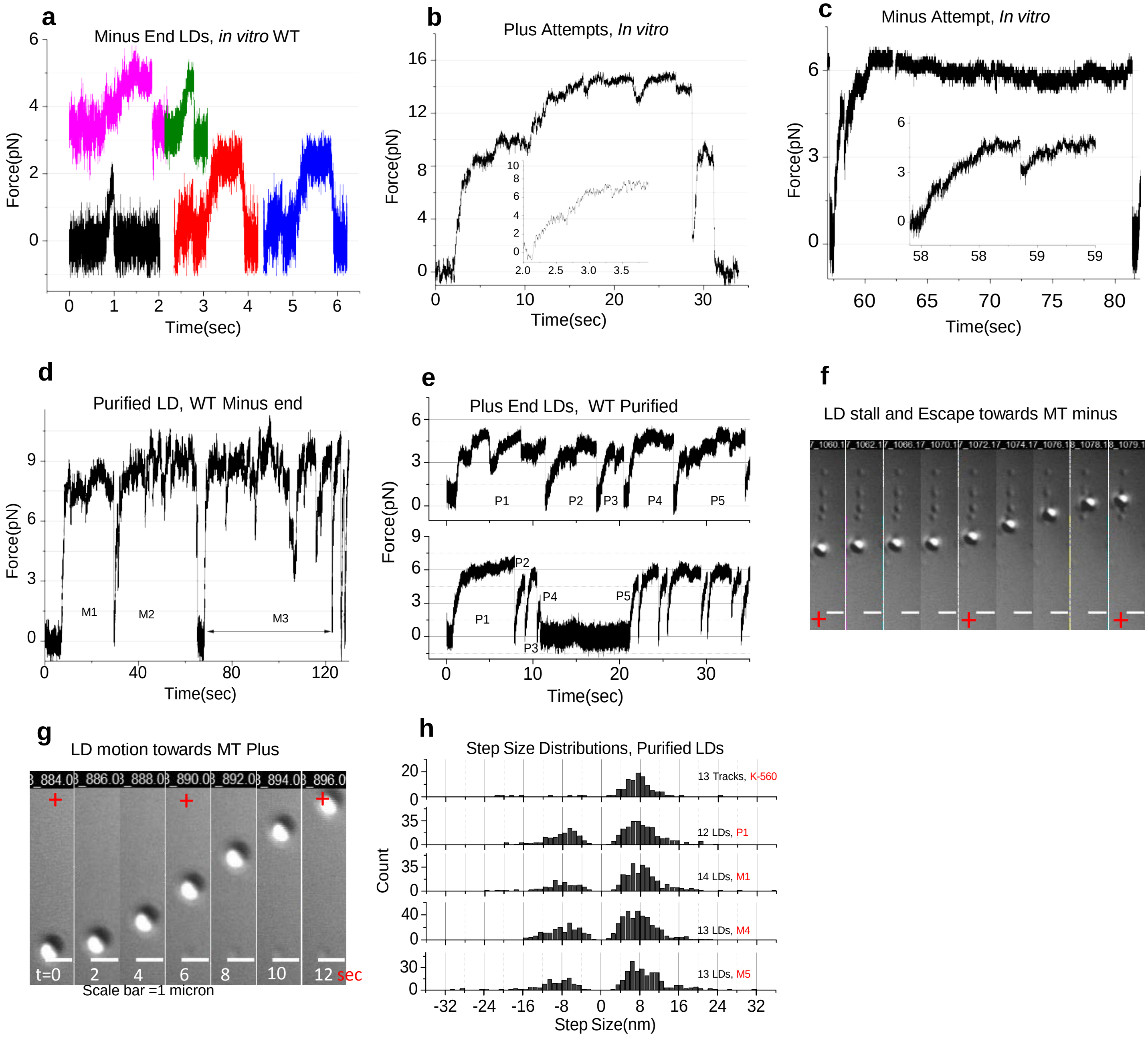

Supplementary Figure 4. Additional traces of purified LDs showing Small forces *and long stalls.* **(a).** Rare minus end stalls of LD showing stalls of about 1 and 2 pN. Long stall of plus end **(b)** and minus end **(c)** moving LDs. Typical minus(**d)** and plus (**e**) traces of purified LDs showing high on-rates and long persistence times (**f** & **g**) Still images of purified LD motion on polarity marked microtubules; showing typical stall and escape of a minus end moving LD(F) and motion towards plus end (**g**) of MT. (**h**) Step detection on single-motor kinesin (top) shows that 8 nm steps are detected when present; such steps are present for plus-end moving LDs (second trace), and for minus-end traces (third through fifth). Very little change occurs between steps detected in M1 and M5 in the purified LDs.

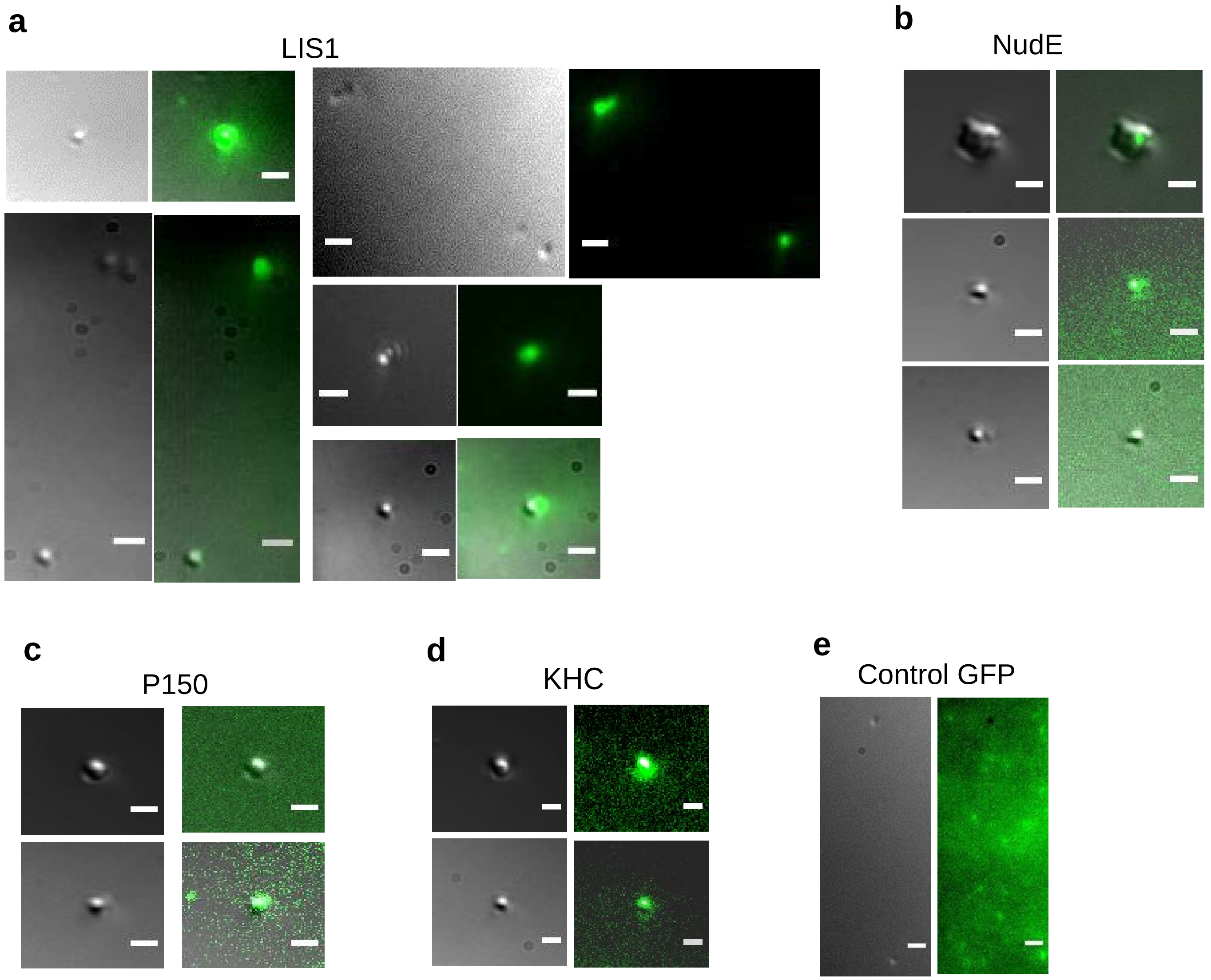

Supplementary Figure 5. TIRFM imaging with primary and fluorescent secondary antibodies against proteins on the purified LDs. Additional DIC (left) and TIRFM (center) images showing the co-localization (right) of LIS1 **(a)**, NudE **(b)**, Dynactin (Only second LD has it) (**c**) and Kinesin Heavy Chain **(d)** on the LDs. (**e**) Control showing no signal. Here anti-GFP ab made in rabbit (same host as LIS1ab) was used to confirm that signal is not due to nonspecific binding of LIS1ab. scale bar=1 micron**.**

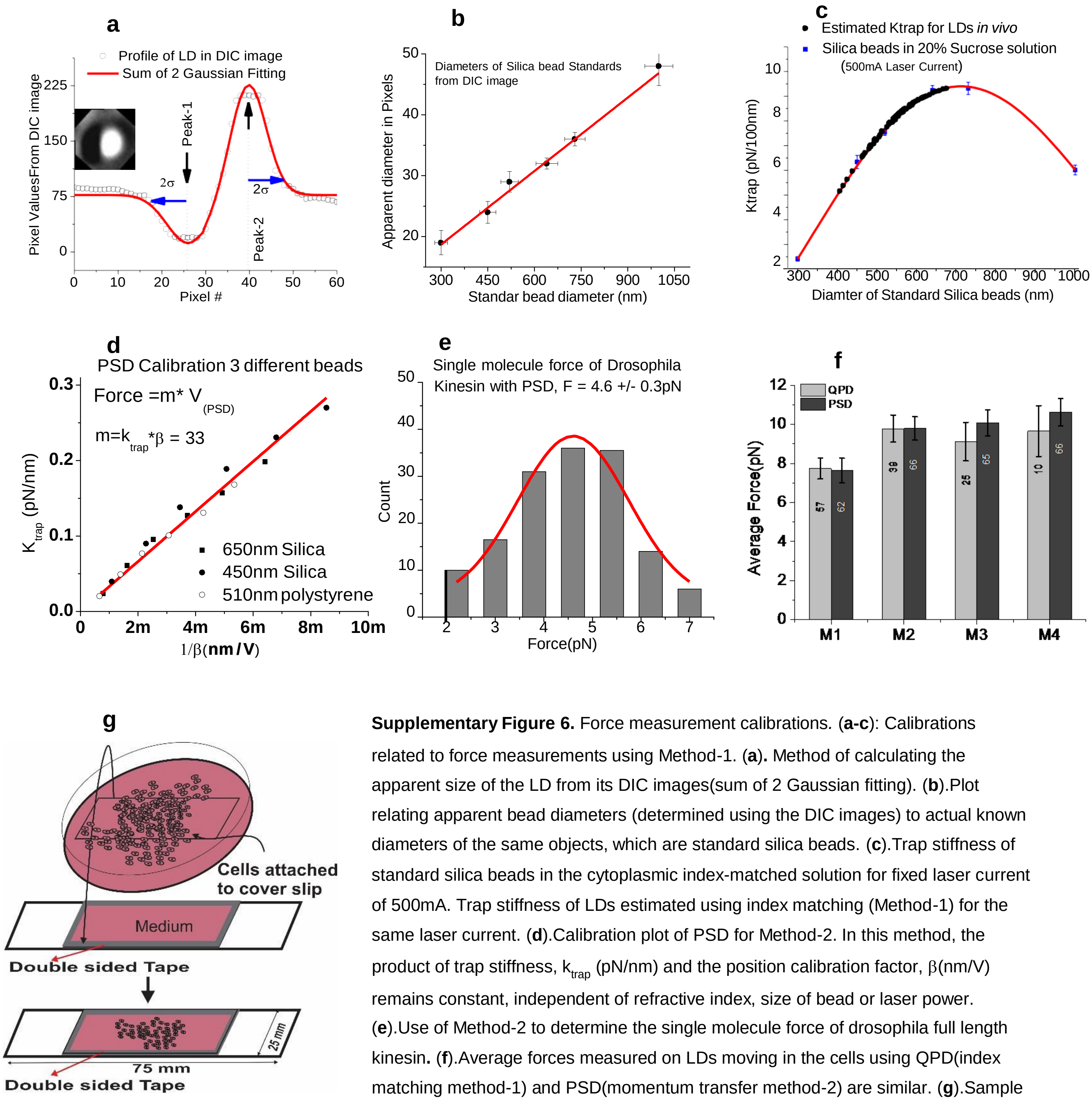

Supplementary Figure 6. Force measurement calibrations. (**a-c**): Calibrations related to force measurements using Method-1. (**a**). Method of calculating the apparent size of the LD from its DIC images(sum of 2 Gaussian fitting). (**b**).Plot relating apparent bead diameters (determined using the DIC images) to actual known diameters of the same objects, which are standard silica beads. (**c**).Trap stiffness of standard silica beads in the cytoplasmic index-matched solution for fixed laser current of 500mA. Trap stiffness of LDs estimated using index matching (Method-1) for the same laser current. (**d**).Calibration plot of PSD for Method-2. In this method, the product of trap stiffness, k_{trap} (pN/nm) and the position calibration factor, β(nm/V) remains constant, independent of refractive index, size of bead or laser power. (**e**).Use of Method-2 to determine the single molecule force of drosophila full length kinesin. (**f**).Average forces measured on LDs moving in the cells using QPD(index matching method-1) and PSD(momentum transfer method-2) are similar. (**g**).Sample chamber constructed to prepare the cells for force measurements.

Supplementary Note 1: Development of a new system to measure forces

One of the challenges in measuring forces *in vivo* using optical trap is calibration of the applied force. We employed two different techniques for this purpose and both were found suitable. The first, a method we previously applied, is based on *in vitro* calibrations using refractive index-matching approach[1], measuring laser deflection with a QPD. The second is a newer method[2-4], detecting momentum-changes in the laser beam at back focal plane of the condenser using a PSD (position sensitive detector) to directly measure the beam deflections. It thus measures force directly (Fig. 1b and Fig. 3a & 3b, Supplementary Fig. S2a-2j, Supplementary Fig. S6d-S6f), and does not need the determination of LD position relative to the trap center, in contrast to the index-matching approach(Supplementary Fig. S6a-S6c). Importantly, because it measures momentum changes of the scattered laser light, Method-2's determination of applied force is insensitive to cargo size and shape, or exact position of the trapped object relative to the trap center. In our measurements both approaches yielded similar results (the average forces measured for LDs are similar, Supplementary Fig. S6f). Note that in either case, because some of the measurements span over 50 seconds, implementation of an independently running focus lock system in our setup eliminated slow drift in the microscope stage.

We use a Nikon TE200 inverted DIC microscope; on which two optical traps were assembled using single mode diode laser (980 nm, 700 mW from EM4 Inc.) and an 830 nm, 1 Watt, Ti-Sapphire laser (Coherent, Verdi-5). An automated xy-piezo-mirror (Madcity labs) is placed in the back focal plane of the objective to steer the optical trap in the field of view by the application of DC voltage (0-10 V, two independent DAC outputs from 16 bit resolution NI card for X and Y motion, capable of corrections down to 1 nm). The calibration factor for steering the trap using mirror was estimated each time by identifying LD positions inside the cell at minimum and maximum voltages. The setup is designed in such a way that with a mouse click in the vicinity of the moving LD in the DIC video, the droplet is first moved (via motorized stage, Ludl Electronics) close to the trap center, and then the trap is positioned with more accuracy using the real time template matching and XY-piezo-mirror. We restricted the piezo-mirror based steering distance of the trap to within few microns from the center of field of view by using linearly moving XY-stage. The range of trap motion using piezo-mirror was calibrated by trapping a freely diffusing LD in the cell each time the coverslip was changed. Analog voltage was incremented in steps of 2 volts from 0-10 V, using 16 bit D/A outputs of NI card. Signal was simultaneously

applied to X and Y channels and LD positions recorded using template matching to obtain the scaling factor. This combination greatly reduced the uncertainty in co-localizing the centers of LD and trap. More importantly, decreased the effective time needed to apply the trapping force after identifying the LD center using real time template matching (Note that we employed LD template matching twice; first, to identify the droplet when it is far away from the trap and second, immediately after the LD is brought to the center of field of view and just before applying the trap). This combination also provides the ability to automatically scan the sample over a wide range and at the same time achieve high accuracy (~10nm) for the trap-droplet relative position. High resolution position information were obtained from either a QPD (Method-1) or a PSD (Method-2, momentum transfer), see Methods. Autofocus lock system was built using an 850nm diode laser (in TIRF mode), a QPD and a piezo z-stage (from PI) on which the sample is mounted.

Supplementary Note 2. Identification of Minus and Plus End Droplets

In principle, there could be local changes to/uncertainty in microtubule orientation. However, we were careful to avoid such complications, because we only analyzed droplets whose motion was approximately perpendicular to the cell periphery, and occurring in locations where the majority of the vesicle traffic was linearly outward from the cell center. Certainly the vast majority of those droplets moving ‘out’ (and that thus fit this criteria) are going to be moving plus-ends, and the vast majority of those moving ‘in’ are towards minus-ends. Ours is a statistical argument—and most of time significantly correct with regard to orientation; an occasional incorrectly identified droplet will not drastically alter our conclusions. There is a large body of work amassed over many years that indicates that in non-epithelial cells, the minus-ends are close to the nucleus, and the plus-ends are close to the periphery. This is also supported by our EB1-GFP and MT imaging (see supplementary movies 11 & 12). Further, the differences between our plus-end data and minus-end data unambiguously show that we can appropriately identify each direction of travel. For instance, pooling all the plus-end forces, and all of the minus-end forces, we did a Kolmogorov-Smirnov test, to compare the distributions, and found that the hypothesis that the two distributions are the same can be rejected with a p value smaller than 0.0000001. Thus, given the extensive work from others in the field establishing that in normal cells such as COS1 the microtubule plus-ends are oriented towards the periphery, combined with the dramatic differences we see in plus-end vs. minus-end motion, there seems little probability that we are in fact unable to differentiate between

the two. Critically, our *in vivo* data is consistent with our *in vitro* studies along polarity-marked microtubules, where there is absolutely no ambiguity as far as directionality of transport: in vitro as well as in vivo, force adaptation occurs by altering minus-end force persistence.

Supplementary Note 3. Criteria for scoring attempts and Escapes.

A successful escape was easily determined, as the LD mostly walked out of the trap. A failed escape attempt was scored by considering both video tracking and high resolution PSD data (2 kHz). The criteria used for scoring a failed attempt is that LD position must be distinctly away from baseline, and that clear detachments should occur, resulting in the LD quickly falling to within 20% of the maximum displacement from the baseline (Note that in majority of the events the detachments brought the LD to within 10-15% of peak displacement in ~ 0.05 sec, See Fig. 1a & 1b, Supplementary Fig. S2a-2j). We had no difficulty in observing the clean detachments in case of LDs possibly because they are not membrane bound. We hypothesize that this may not be true in membrane bound vesicles as the membrane can in principle act as an elastic tether thus making it difficult to observe clean detachments. In these measurements the clean detachments could be easily identified by the abrupt change or discontinuity in the slope of the LD track both in the PSD and video data.

Errors in escaped fraction (*f*) were determined as $\sqrt{\text{[illegible]}}$ for *n* droplets tested.

Supplementary Note 4: On the apparent increase in escape probability on P2 in the P150 siRNA background. While an increase in plus-end escape probability was observed for the second plus-end escape in the P150 siRNA background (see Fig. 2f), we view this as a statistical fluke. We base this on two observations. First, in additional repeated experiments with P150 RNAi cells, the escape probability for the second plus attempt was not observed to increase, although the minus end escape probabilities went up as found earlier. (44 LDs tested in the repeat experiment). The results were combined, which brought down the p-value (to 0.06), but we acknowledge it is still rather large. Second, in addition to the escape probability measurements, we also carried out high-force measurements. In these more quantitative measurements, in the P150 RNAi background, there was no increase in P2 maximum force or duration of force production (see Fig. **3b, 3d**), again consistent with the hypothesis that the increased P2 escape seen in Fig. 2f was a statistical

fluke. Errors in escaped fractions in each attempt (*f*) were estimated with $\sqrt{\quad}$ [illegible] for *n* droplets that made the escape attempt.

Supplementary Note 5. Labile Microtubules are unlikely to explain the Adaptation

In the peripheral region where we make measurements we did not detect obvious microtubule motion (see supplementary movie 12). We can eliminate buildup of sustained microtubule deformations as key contributors to adaptation, because of the long (10-second) periods between attempts, where no force is required to keep the droplet positioned in the trap (see e.g. Fig. 1a & 1b, Supplementary Fig. S2a-S2b, S2e-S2j); during such periods any putative microtubule deformations would relax. Further, we also see the time dependent systematic increase in the force persistence of motor complexes with purified LDs *in vitro,* in the absence of labile microtubules. Thus, adaptation results from changes in the function of the machinery on the cargos themselves, rather than motion of the microtubules. Ultimately, the machinery driving minus-end directed lipid-droplet transport adapts to opposition to motion, and this adaptation requires the combined use of Dynactin, LIS1, and NudE & NudEL. Such a conclusion is consistent with the in vitro experiments which still have force adaptation, though they occur along static taxol-stabilized microtubules stuck to glass coverslips.

Supplementary Note 6. Step sizes of purified LDs under load

Since dynein can take different step sizes[5], and dynactin can change dynein's step-size distribution[6], we wondered what happened to step size distributions during adaptation. We first tested our analysis on polystyrene beads driven by single kinesin-1 motors (K-560, Supplementary Fig. S4h, top), confirming that 8 nm steps were detected as expected. Then, we examined stepping behavior of plus-end moving LDs *in vitro* (Supplementary Fig. S4h, second). While forward motion was similar, with predominantly 8 nm steps detected, more back-steps were detected. With this calibration done, we examined minus-end motion. On, average step size distributions (Supplementary Fig. S4h) for attempts M1 (n=14), M4 (n=13) and M5 (n=13) were similar, and were centered around 8nm with perhaps a hint of a small peak at 16nm. We found no significant change in step size distributions during the course of minus end adaptation. While the data do not directly support the hypothesis that adaptation involves alteration of dynein step sizes, we should note that the cargo motion here

involves multiple dynein motors working together, and average motion of the cargo's center of mass may not sensitively reflect stepping dynamics of individual motors in the ensemble.

Method: PSD data (2 kHz) of purified LD's escape attempts in the trap (k_{trap} ~ 6pN/100nm) was analyzed using custom written MATLAB code reported in ref[6]. Data was pooled from multiple traces of M1 (n=14), M4 (n=13), M5 (n=13), & P1 (n=12 L) to generate plots of distributions. Analysis of *in vitro* purified truncated kinesin-1 traces (n=13) in the trap (Kinesin-560 attached to 500 nm polystyrene beads, K_{trap}=5 pN/100nm) yielded a peak centered at 8 nm with negligible back steps, thus validating the method.

Supplemental References

1. Shubeita, G.T. *et al.* Consequences of motor copy number on the intracellular transport of kinesin-1-driven lipid droplets. *Cell* **135**, 1098-1107 (2008).
2. Farre, A., Marsa, F. & Montes-Usategui, M. Optimized back-focal-plane interferometry directly measures forces of optically trapped particles. *Optics express* **20**, 12270-12291 (2012).
3. Tolic-Norrelykke, S.F. *et al.* Calibration of optical tweezers with positional detection in the back focal plane. *Rev Sci Instrum* **77** (2006).
4. Jun, Y.G., Tripathy, S.K., Narayanareddy, B.R.J., Mattson-Hoss, M.K. & Gross, S.P. Calibration of Optical Tweezers for In Vivo Force Measurements: How do Different Approaches Compare? *Biophysical journal* **107**, 1474-1484 (2014).
5. Rai, A.K., Rai, A., Ramaiya, A.J., Jha, R. & Mallik, R. Molecular adaptations allow dynein to generate large collective forces inside cells. *Cell* **152**, 172-182 (2013).
6. Tripathy, S.K. *et al.* Autoregulatory mechanism for dynactin control of processive and diffusive dynein transport. *Nature cell biology* **16**, 1192-1201 (2014).
